# From Corn Gluten Meal to Bioactive Glutamine Peptides: Stepwise Enzymatic Release, Peptidomics Analysis, and Identification of a Novel Peptide QFSLP Alleviates LPS-Induced Inflammation

**DOI:** 10.3390/foods15162904

**Published:** 2026-08-19

**Authors:** Guanlong Li, Xiaolan Liu, Zhengfei Miao, Yuhao Zhao, Quanxin Wang, Xiqun Zheng

**Affiliations:** 1 Heilongjiang Provincial Key Laboratory of Corn Deep Processing Theory and Technology, College of Food and Bioengineering, Qiqihar University, Qiqihar 161006, China; 03580@qqhru.edu.cn (G.L.); wangquanxin979@163.com (Q.W.); 2 Engineering Research Center of Plant Food Processing Technology, Ministry of Education, Qiqihar 161006, China; 3 College of Food Science, Heilongjiang Bayi Agricultural University, Daqing 163319, China

**Keywords:** corn gluten meal, glutamine peptide, molecular docking, intestinal inflammation, Caco-2 cell

## Abstract

Glutamine peptides not only serve as delivery vehicles for supplemental glutamine but also frequently possess unique biological activities that surpass glutamine itself, playing a crucial role in maintaining intestinal health. Corn gluten meal, as a major byproduct of corn processing with substantial production volume, is rich in glutamine, making it an ideal raw material for preparing glutamine peptides. This study aims to establish an enzymatic hydrolysis process for the efficient release of glutamine peptides from corn gluten meal and to identify glutamine peptides with gut health-maintaining effects. The results indicate that stepwise enzymatic hydrolysis of corn gluten meal using Protamex and Trypsin yields a glutamine-rich corn protein hydrolysate (GRCH). A total of 175 glutamine peptides were further identified from the low-molecular-weight fraction of GRCH. Through physicochemical property analysis and molecular docking technology, five glutamine peptides with potential inhibitory activity against the JAK2/STAT3 signaling pathway were screened, among which QFSLP demonstrated outstanding performance. In vitro experiments demonstrate that QFSLP is fully absorbed by intestinal epithelial cells and effectively alleviates LPS-induced inflammatory responses in intestinal cells. Following QFSLP intervention, the levels of proinflammatory factors TNF-α, IL-1β, and IL-8 in cells were significantly reduced (*p* < 0.05), while the level of the anti-inflammatory factor IL-10 was significantly increased (*p* < 0.05). The findings of this study contribute to the industrial-scale production of glutamine peptides and provide experimental evidence for their development in gut health-related products.

## 1. Introduction

Glutamine (Gln, Q) is the most abundant amino acid in the human body, playing a role in maintaining immune function, nitrogen balance, and intestinal health [1]. As a conditionally essential amino acid, the demand for glutamine surges under metabolic stress caused by injury or illness to support cellular repair and regeneration processes [2]. Although Gln is an effective nutritional supplement, its monomer exhibits low solubility in water and readily produces toxic substances such as pyroglutamic acid under thermal conditions, limiting its application in health-demand scenarios [3]. Compared to Gln monomers, glutamine peptides demonstrate significant advantages. The Gln residue in the peptide chain is more stable due to the protection of adjacent amino acids, while the peptide molecule generally exhibits better solubility than its monomeric form. Moreover, in addition to serving as Gln delivery vehicles, some glutamine peptides themselves possess unique biological activities that extend beyond those of Gln [4]. Therefore, shifting the research focus to glutamine peptides represents a more promising strategy.

Glutamine peptides typically refer to short peptides containing Gln residues. Similar to Gln monomers, they play a crucial role in maintaining intestinal barrier integrity and alleviating inflammation. Collagen peptides GPSGPQGSR demonstrate superior efficacy compared to Gln monomers in repairing TNF-α induced intestinal barrier dysfunction in the Caco-2 cell model [5]. The glutamine-rich corn protein hydrolysate APH significantly improves LPS-induced intestinal barrier dysfunction in Caco-2 cells and alleviates inflammatory responses by regulating inflammatory factor levels. Its mechanism involves modulating tight junction protein expression and inhibiting the activation of NF-κB and MLCK signaling pathways [6]. The extensively studied glutamine dipeptide Ala-Gln not only breaks down in the human body to replenish Gln but also possesses intestinal protective functions that extend beyond mere nutritional supplementation. Ala-Gln significantly alleviates DSS-induced acute colitis in mice. Its intervention inhibits the activation of the PI3K-Akt/NF-κB/STAT3 inflammatory signaling pathways in the colon and improves the composition of the gut microbiota [7]. Additional studies indicate that Ala-Gln supplementation enhances the Wnt/β-catenin signaling pathway, thereby accelerating stem cell-mediated small intestinal epithelial development and conferring greater regenerative capacity to the intestinal epithelium [8]. It is evident that peptides containing glutamine hold greater potential for development in the functional food sector, and their physiological functions are multifaceted.

The primary methods for obtaining glutamine peptides currently are chemical synthesis and enzymatic hydrolysis [9]. Chemical methods, represented by solid-phase synthesis, enable the precise synthesis of high-purity peptides with specific sequences, making them the preferred choice for fundamental research and the synthesis of high-value bioactive peptides. However, its harsh reaction conditions (strong acids, strong bases, and complex reagents with potential toxicity) and high cost make it difficult to meet the requirements of the food industry for safety, economy, and large-scale production [10]. In contrast, enzymatic hydrolysis offers mild conditions, environmental safety, and controllable costs, making it more advantageous for industrial-scale production of glutamine peptides from glutamine-rich plant proteins. Nevertheless, the release of peptides from proteins is influenced by numerous factors, including the structure of the substrate protein, the specificity of the protease, and variations in enzymatic conditions, resulting in the enzymatic products not being entirely effective [11]. Therefore, it is necessary to identify suitable enzymatic hydrolysis processes to guide the resulting peptide profile to some extent and enhance the yield of target peptide components.

Corn gluten meal (CGM) is the primary byproduct of wet corn starch production, with a protein content of 60~70% [12]. Corn is widely cultivated worldwide, with China producing over 100 million tons annually. Approximately 17% of this crop is processed into starch, generating millions of tons of CGM [13]. Corn protein is rich in approximately 16% glutamine [14]; furthermore, the substantial yield of CGM makes it an ideal raw material for the large-scale production of glutamine peptides. Additionally, in the primary amino acid sequence structure of corn protein, hydrophobic amino acids are often found near Gln (α-Zein, P04703, https://www.uniprot.org/uniprotkb/P04703/entry (accessed on 19 July 2026); Glutelin, P04706, https://www.uniprot.org/uniprotkb/P04706/entry (accessed on 19 July 2026)). This unique sequence structure endows glutamine peptides derived from corn protein with potential biological activity. Compared to glutamine monomers and glutamine peptides from other sources, glutamine peptides derived from corn protein may exhibit superior biological activity. Based on this, in this study, CGM was used as the raw material. According to the position of glutamine in the primary structure of corn protein, a stepwise enzymatic hydrolysis strategy targeting the efficiently releasing glutamine peptides was established, and glutamine-rich corn protein hydrolysate (GRCH) was prepared. Through LC-MS/MS combined with computer-assisted screening, potential bioactive glutamine peptides were obtained, and their ability to alleviate inflammation in LPS-induced Caco-2 cells was further demonstrated.

## 2. Materials and Methods

### 2.1. Materials and Chemicals

CGM (60% *w*/*w*) was purchased from Longjiang Fufeng Biotechnology Co., Ltd. (Qiqihar, China). Protamex (38,000 U/g), Neutrase (21,000 U/g), Flavourzyme (23,000 U/g) and Papain (31,000 U/g) were obtained from Novo Nordisk (Bagsvaerd, Denmark). Trypsin (31,000 U/g) was provided by Lanji Technology Development Co., Ltd. (Shanghai, China). Sephadex G-25, Dulbecco modified Eagle medium (DMEM), and fetal bovine serum (FBS) were from GE Healthcare (Pittsburgh, PA, USA). Caco-2 cells were purchased from JianCheng Co., Ltd. (Nanjing, China). LPS was purchased from Macklin (Shanghai, China). All other reagents are of analytical grade.

### 2.2. Preparation of Corn Protein Hydrolysates with High Glutamine Content

CGM underwent pretreatment based on our team’s prior research, including extrusion and starch removal, to enhance the purity and modification of protein, and improve its enzymatic hydrolysis efficiency [15]. Prepare the pretreated CGM into a 10% substrate solution, adjusting the pH of the substrate solution using NaOH or HCl. The first step of enzymatic hydrolysis is carried out using Protamex under the conditions as shown in Table 1. After the first enzymatic digestion step, adjust the solution temperature and pH to the conditions for the second enzymatic digestion step. The second step involves enzymatic digestion using Neutrase, Flavourzyme, Papain, and Trypsin (as shown in Table 1). After the enzymatic digestion is completed, the enzymatic digestion solution is heated in boiling water to inactivate the enzymes for 10 min. Then, it is centrifuged at 4500 r/min for 15 min. Finally, the supernatant is collected for freeze-drying treatment.

### 2.3. Optimization of Enzymatic Conditions

Based on enzyme screening results, the enzymatic digestion conditions for Trypsin in the P + T combination were optimized using a single-factor method. The ranges for these conditions were as follows: enzyme addition (200–1000 U/g), enzymatic hydrolysis temperature (30–50 °C), enzymatic hydrolysis time (1–3 h), and enzymatic hydrolysis pH (6.5–8.5). Based on the results of single-factor optimization, a Box–Behnken optimized experiment was designed. The independent variables were enzymatic hydrolysis time, enzymatic hydrolysis temperature, and enzyme addition, with Gln content as the response variable. The experimental design is shown in Table 2 and Table 3. Based on the finally optimized enzymatic hydrolysis conditions, a glutamine-rich corn protein hydrolysate (GRCH) was obtained.

### 2.4. Determination of Hydrolysis Degree

The degree of hydrolysis (DH) is calculated using the pH-stat method [16], with the formula as follows:

(1) $$\mathrm{DH}(\%)=\frac{V\times N}{M_{P}\times \mathrm{htot}\times \alpha }\times 100$$

(2) $$\alpha =\frac{10^{pH-pK}}{1+10^{pH-pK}}$$

In the equation, V represents the volume of NaOH solution consumed during the enzymatic hydrolysis process (mL); N represents the molar concentration of the NaOH solution (mol/L); Mp denotes the total protein mass in the sample (g); htot represents the total number of peptide bonds per unit protein mass (mmol/g, with 8.38 used for corn protein); α indicates the dissociation degree of α-amino acids; pH denotes the initial reaction pH; and pK represents the dissociation constant of α-amino acids (assumed as 7.0).

### 2.5. Determination of Gln Content

Determination of Gln content used the BTI protection method [17]. Add 0.5 mL of sample solution (100 mg/mL), 2 mL of bis-1,1-trifluoroacetoxy-iodobenzene (BTI) in acetonitrile–water solution (10 mg/mL), and 0.5 mL of pyridine solution (50 μmol/mL) to an ampoule. Mix and react at 50 °C for 2 h. This step is performed to protect the Gln in the sample. The sample and the unprotected sample were subjected to acid hydrolysis separately. Add 5 mL of 6 mol/L HCl to the ampoules containing each sample, seal the ampoules under vacuum, and hydrolyze at 110 °C for 24 h. After acid hydrolysis is complete, collect the samples and adjust the pH to 6–8 using 6 mol/L NaOH. Finally, glutamate content was measured using the S-10 biosensor analyzer (Sieman, Shenzhen, China). The difference in glutamate levels between the two samples represents the Gln content.

### 2.6. Ultrafiltration of GRCH

GRCH was ultrafiltrated using the HYM-Multi-RN multifunctional laboratory separator (Ge, Pittsburgh, PA, USA). Ultrafiltration membranes with molecular weight cutoffs of 3 kDa and 5 kDa were selected to fractionate the sample into three components: GRCH-1 (>5 kDa), GRCH-2 (3–5 kDa), and GRCH-3 (<3 kDa). These fractions were collected, lyophilized, and stored for subsequent use.

### 2.7. Determination of Antioxidant Indicators In Vitro

Prepare a series of samples with concentration gradients, determine the antioxidant capacity of the samples using the following method, and finally calculate the IC_50_ value for the antioxidant capacity of the samples.

The hydroxyl radical scavenging capacity of the sample was determined using a modified version of the method described by Chong et al. [18]. Mix 2 mL of sample, 2 mL of FeSO_4_ solution (6 mmol/L), and 2 mL of H_2_O_2_ solution (2 mmol/L) thoroughly. Allow the mixture to stand for 10 min, then add 2 mL of salicylic acid solution (6 mmol/L). Incubate the mixture at 37 °C for 30 min. Finally, measure the absorbance at 510 nm. Distilled water was used to replace salicylic acid and the sample, respectively, as the control group and blank group. The calculation formula is as follows:

(3) $$\mathrm{Hydroxyl}\;\mathrm{radical}\;\mathrm{scavenging}\;\mathrm{activity}\;=(1-\frac{A_{1}-A_{2}}{A_{0}})\times 100\%$$

In the given equation, A_0_ corresponds to the absorbance of the blank, A_1_ to the sample, and A_2_ to the control group.

The ABTS radical scavenging capacity of the samples was determined using the method of Zhang et al. with modifications [19]. Mix equal volumes of 7 mmol/L ABTS solution and 2.45 mmol/L potassium persulfate solution, and incubate in the dark for 12–16 h to obtain the ABTS radical solution. Dilute the ABTS radical solution with PBS buffer to achieve an absorbance value of 0.7 ± 0.02 at 734 nm. Add 0.1 mL of sample solution and 0.1 mL of diluted ABTS radical solution to a 96-well plate. Incubate in the dark for 10 min, then measure the absorbance at 734 nm. Use PBS buffer solution as the control group instead of the sample solution. The calculation formula is as follows:

(4) $$\mathrm{ABTS}\;\mathrm{radical}\;\mathrm{scavenging}\;\mathrm{activity}\;=(1-\frac{A_{1}}{A_{0}})\times 100\%$$

In the equation, A_0_ represents the absorbance of the control group, and A_1_ represents the absorbance of the sample group.

The Fe^2+^ chelation capacity of the sample was determined using a modified version of the method described by Wang et al. [20]. Mix 2.5 mL of the sample solution, 0.1 mL of FeSO_4_ solution (2 mmol/L), and 0.2 mL of phthalazine solution (2 mmol/L). Incubate for 10 min, then measure the absorbance at 562 nm. Distilled water was used to replace FeSO_4_ and the sample, respectively, as the control group and blank group. The calculation formula is as follows:

(5) $${\mathrm{Fe}}^{2+}\;\mathrm{chelation}\;\mathrm{capacity}\;=(1-\frac{A_{1}-A_{2}}{A_{0}})\times 100\%$$

In the equation, A_0_ represents the absorbance of the blank group, A_1_ represents the absorbance of the sample group, and A_2_ represents the absorbance of the control group.

### 2.8. Identification of Peptide Sequences

First, GRCH-3 was separated using Sephadex G-25 gel chromatography. Load GRCH-3 onto a Sepadelex G-25 column equilibrated with 0.02 mol/L PBS buffer (containing 0.15 mol/L NaCl) and elute at a flow rate of 5 mL/min. Based on the standard protein elution profile and molecular weight standard curve, collect the fraction of GRCH-3 with a molecular weight less than 1 kDa. Bioteach Pack Co., Ltd. (Beijing, China) was commissioned to identify peptide sequences in the intercepted fraction using Nano LC-MS/MS.

### 2.9. Analysis of the Physicochemical Parameters of Peptide Sequences

Using the “peptides” tool in R and the ExPASy-ProtParam tool, we analyzed selected physicochemical parameters of the identified peptide sequences, including net charge, Boman index, instability index, aliphatic index, isoelectric point, hydrophobicity, hmoment α-helix, and hmoment β-sheet.

The toxicity of the identified peptides was predicted using the ToxinPred web server (https://webs.iiitd.edu.in/raghava/toxinpred/, accessed on 19 July 2026). All peptides were submitted in FASTA format with default parameters. Peptides predicted as “Non-toxin” were considered to have a low toxicity risk and were retained for subsequent experimental validation.

### 2.10. Molecular Docking of Peptide Sequences with JAK2 and STAT3

Perform molecular docking between the peptide sequence and the receptor protein using the method described by Ge et al. [21]. Obtain the crystal structures of JAK2 (PDB ID: 3KRR) and STAT3 (PDB ID: 6NJS) from the RCSB Protein Data Bank. Using Pymol software, remove water molecules and ligands from the JAK2 and STAT3 structures while adding polar hydrogen atoms to obtain receptor structures suitable for docking.

Since the binding modes of short glutamine-containing peptides to JAK2 and STAT3 have not been experimentally characterized, a blind docking strategy was employed. Grid boxes with dimensions of 80 × 80 × 80 Å and a spacing of 0.375 Å were centered on the coordinates of JAK2 (x: 15.21, y: 11.247, z: 4.171) and STAT3 (x: 13.498, y: 54.118, z: 0.1), respectively, to encompass the entire protein surface and allow unbiased sampling of all potential interaction sites.

The three-dimensional structures of the linear peptides were initially constructed using ChemDraw 3D 8.0, with all amino acid residues specified in the L-configuration. Each peptide was built as an extended chain and then subjected to energy minimization in AutoDock Tools 4.2.6 using the MMFF94 force field to obtain a single low-energy conformer for subsequent docking. It should be noted that short linear peptides in solution do not adopt a single fixed conformation but rather exist as an ensemble of rapidly interconverting conformers. The present study employed a single energy-minimized conformer per peptide as a pragmatic screening approach; the resulting binding poses should therefore be interpreted as preliminary and do not capture the full conformational flexibility of these peptides.

Molecular docking was performed using AutoDock software 4.2.6 within the defined grid boxes. Following docking, Discovery Studio was used to analyze and visualize the interactions between each peptide and the receptor proteins. The top-ranked binding poses were selected based on binding energy for further inspection.

### 2.11. The Synthesis of the Identified Peptide QFSLP

The QFSLP monomer was commissioned to be synthesized by QiangYao China Peptides Co., Ltd. (Shanghai, China), and the purity of the QFSLP monomer was ≥98% (Figure S1).

### 2.12. Caco-2 Cell Culture

Caco-2 cells were cultured in Dulbecco’s Modified Eagle Medium (DMEM) supplemented with 10% (*v*/*v*) fetal bovine serum (FBS) and 1% (*v*/*v*) penicillin–streptomycin. Cells were passaged when confluence reached 80–90%. Cells from the 20th to 30th passage were used for experiments.

### 2.13. Caco-2 Cell Monolayer Transport Assay

Follow the method described by Jing et al. to establish a Caco-2 cell monolayer model and perform a transport assay [6]. Seed Caco-2 cells at a concentration of 2 × 10^5^ cells/mL onto the apical side (AP side) of a 6-well Transwell plate. Add complete medium to the basolateral side (BL side) and culture for 14 days until cell differentiation occurs. The TEER value of cells was measured using a Millicell ERS ohmmeter (Millipore, Boston, MA, USA). When the TEER value stabilized above 800 Ω·cm^2^, the Caco-2 cell monolayer was considered to have formed. Prior to the transport assay, wash the Caco-2 cell monolayer with Hank’s buffer. Then add 0.5 mL of QFSLP–Hank’s solution (10 mg/mL) to the AP side and 1.5 mL of Hank’s buffer to the BL side. After incubating for 2 h at 37 °C and 5% CO_2_, collect the BL side samples for mass spectrometry sequencing.

### 2.14. Establishment of the Caco-2 Cell Inflammatory Model and Determination of Inflammatory Marker Levels

Caco-2 cells were seeded at a concentration of 2 × 10^5^ cells/mL in a 6-well plate. The medium was changed every other day, and the cells were cultured for 14 days. Cells were treated with 100 μg/mL QFSLP for 24 h. Subsequently, without removing the peptide-containing medium, LPS (10 μg/mL final concentration) was added directly to the culture wells, and incubation continued for an additional 24 h. The normal control group received an equivalent volume of serum-free medium at both time points over the total 48 h period. The LPS model group received serum-free medium for the first 24 h and LPS for the subsequent 24 h. All groups were processed simultaneously to minimize inter-assay variability. Supernatants were collected, and LDH activity was measured using an LDH assay kit (Keqiao Biotechnology, Shanghai, China). Cells were harvested and lysed, and levels of TNF-α, IL-1β, IL-8, and IL-10 were determined using ELISA kits (Keqiao Biotechnology, Shanghai, China).

### 2.15. Statistical Analysis

All results were expressed as mean ± standard deviations (SD), and statistical comparisons were performed using one-way analysis of variance and Duncan’s test. SPSS Statistics 19 was used to analyze the data. Significant differences were considered at *p* < 0.05. All hydrolysis experiments were conducted in triplicate (*n* = 3) under each set of conditions. All cell-based experiments were performed in at least three independent biological replicates (*n* = 3). Within each biological replicate, six technical repeats were conducted.

## 3. Results and Discussion

### 3.1. Preparation of GRCH

#### 3.1.1. Protease Screening

The CGM mainly consists of glutelin and zein, which are the main storage proteins in corn. In the primary sequence structure of corn protein, Gln is mainly located inside the polypeptide chain, frequently linked to amino acids such as serine, arginine, and leucine. Examples include SQQQQL_150–155_ and RAQQLQQLV_91–99_ in α-zein (P04703, https://www.uniprot.org/uniprotkb/P04703/entry, accessed on 19 July 2026), and glutelin (P04706, https://www.uniprot.org/uniprotkb/P04706/entry, accessed on 19 July 2026) at LQSI_176–179_, RPQPHPQP_91–98_, and RYQA_167–170_. Given that the amide bond of glutamine is easily broken under harsh enzymatic hydrolysis conditions, the use of mild proteases often fails to achieve high DH. To resolve this contradiction, based on the unique amino acid sequence structure of corn protein, this study employs a dual-enzyme stepwise hydrolysis strategy. First, corn protein undergoes preliminary hydrolysis using the relatively mild Protamex, which disrupts its higher-order structure to generate larger molecular weight peptide segments. Additionally, Protamex can hydrolyze leucine sites, releasing peptide sequences containing Gln, which also contributes to the higher Gln content in Protamex corn protein hydrolysates. This result has been confirmed in previous studies [22]. The enzymatic hydrolysis conditions for Protamex have been optimized, yielding DH of 8.29 ± 0.15% and Gln content of 6.7 ± 0.1%. Building upon this foundation, the second step involves selecting a protease with distinct substrate specificity from Protamex for deep hydrolysis, significantly enhancing both the hydrolysis efficiency and the yield of target peptides. Building upon Protamex, this study designed four enzyme combinations capable of complementary digestion with Protamex: Protamex + Neutrase (P + N), Protamex + Flavourzyme (P + F), Protamex + Papain (P + P), and Protamex + Trypsin (P + T). The synergistic effects of these four dual-enzyme combinations were systematically investigated.

**Figure 1:**
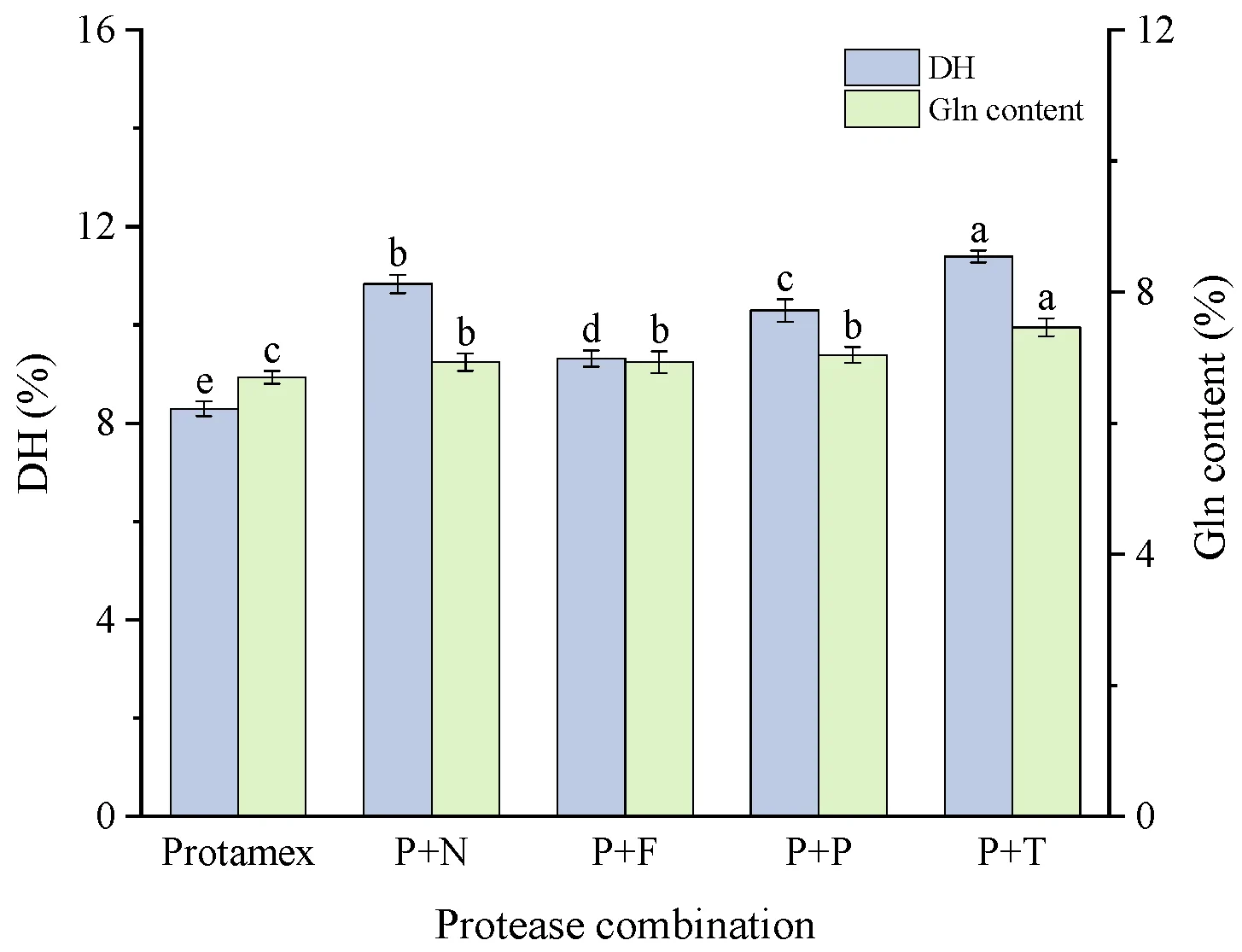
Effects of various enzyme combinations on the DH and Gln content of the hydrolysate. Different lowercase letters represent significant difference (*p* < 0.05).

The test results are shown in Figure 1. The contents of DH and Gln in the hydrolysates prepared by the four dual-enzyme combinations were significantly higher than those in the hydrolysates prepared by a single enzyme (*p* < 0.05). Significant differences were observed among the four dual-enzyme combinations in terms of DH (*p* < 0.05), with the order of DH being P + T > P + N > P + P > P + F. In terms of Gln content, the P + T combination exhibited the highest level, significantly higher than other combinations (*p* < 0.05), while no significant differences were observed among the other combinations (*p* > 0.05). Trypsin acts on arginine residues within peptide sequences. In the primary structure of corn protein, the presence of arginine near glutamine enables trypsin to achieve displaced cleavage when combined with Protamex. This may be the primary reason for the increased glutamine content in the P + T combination. Furthermore, previous studies have demonstrated that proteins with a high Gln proportion exhibit significant resistance to gastrointestinal digestion [23]. This may enable hydrolysates to retain more Gln for intestinal absorption after undergoing gastrointestinal digestion. Overall, the hydrolysate produced by the P + T enzyme combination exhibited the highest DH (11.39 ± 0.12%) and Gln content (7.64 ± 0.09%), indicating that the P + T combination is the optimal enzymatic combination for preparing glutamine-rich corn protein hydrolysate.

#### 3.1.2. Single-Factor Experiment

**Figure 2:**
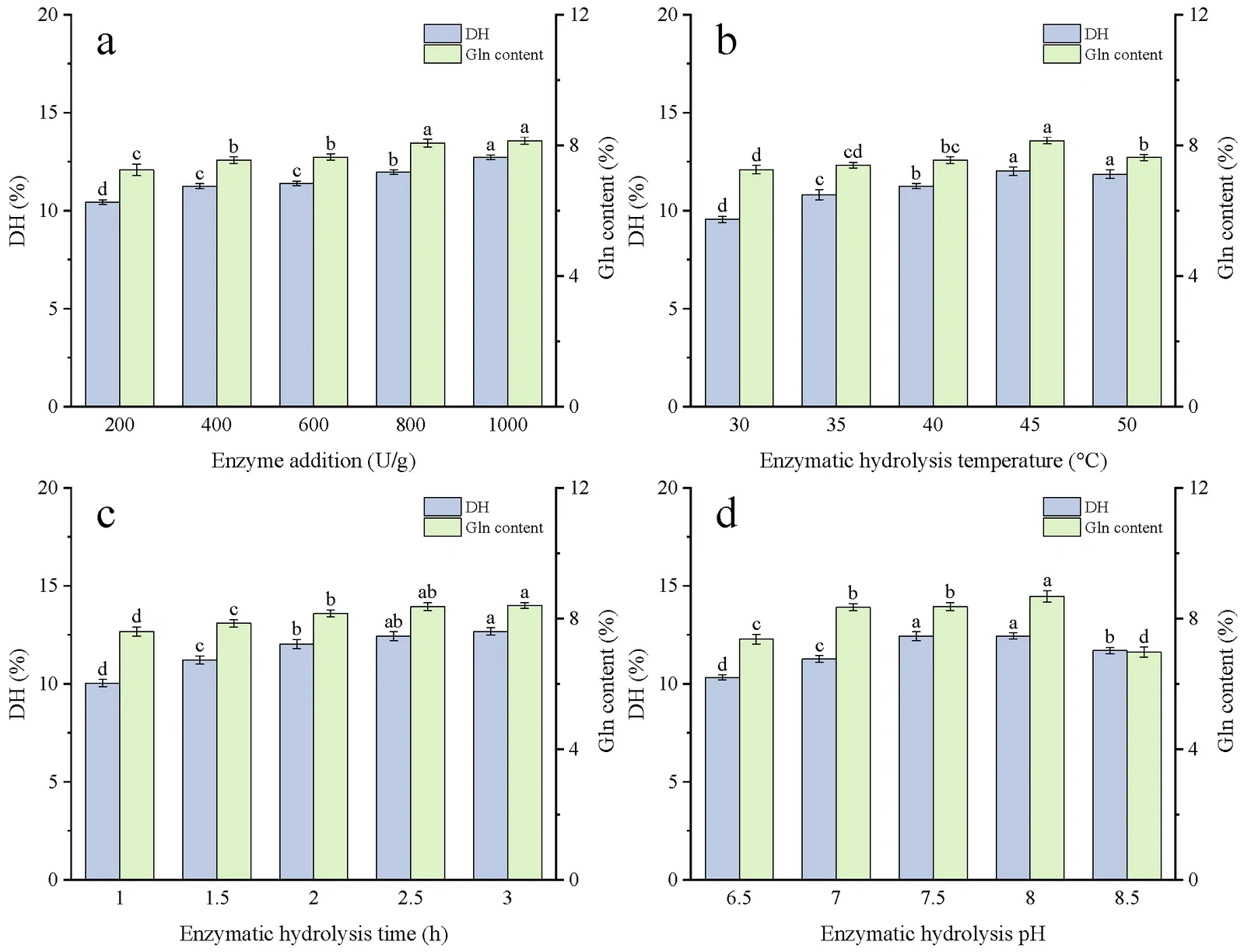
Influence of single factors on the DH and Gln content of the hydrolysate. (**a**) Enzyme addition, (**b**) Enzymatic hydrolysis temperature, (**c**) Enzymatic hydrolysis time, (**d**) Enzymatic hydrolysis pH. Different lowercase letters represent significant difference (*p* < 0.05).

First, the enzyme addition was optimized. As shown in Figure 2a, the DH and Gln content of the hydrolysate increased with rising enzyme addition, reaching a maximum at 1000 U/g. DH represents the extent of peptide bond cleavage in proteins during enzymatic hydrolysis and reflects the yield of hydrolysates [24]. Elevated DH may indicate increased production of glutamine peptides. Test results indicate that hydrolysates prepared with high enzyme addition exhibit the highest levels of DH and Gln. However, since Trypsin is an animal-derived protease with relatively high costs, it is necessary to control its usage to reduce production expenses [25]. When enzyme addition was 400 U/g, the DH (11.26 ± 0.13%) and Gln content (7.55 ± 0.1%) of the hydrolysate reached relatively high levels, showing no significant difference compared to enzyme addition at 600 U/g (*p* > 0.05). After comprehensive consideration, enzyme addition of 400 U/g was selected for subsequent experiments.

As shown in Figure 2b, with increasing enzymatic hydrolysis temperature, the DH and Gln content in the hydrolysate first increased and then decreased, reaching a peak at 45 °C. At this temperature, the DH and Gln content in the hydrolysate were 12.03 ± 0.23% and 8.15 ± 0.1%, respectively. Temperature is one of the key factors affecting protease activity. At the optimal temperature, protease activity reaches its highest level. When temperature changes, whether increasing or decreasing, protease activity will decrease. When temperature changes, the activity of proteases will decrease [26]. Therefore, in subsequent experiments, the enzymatic hydrolysis temperature was selected as 45 °C.

The effect of enzymatic hydrolysis time on the properties of the hydrolysate is shown in Figure 2c. At 3 h, the hydrolysate exhibited the highest DH and Gln content. As the enzymatic hydrolysis time increases, the levels of DH and Gln first rise and then plateau. This trend may be attributed to the initial stage of the hydrolysis reaction, where substrates are continuously degraded by proteases, leading to rapid product accumulation. However, as hydrolysis time extends, the decreasing substrate concentration causes the reaction rate to gradually slow down, ultimately stabilizing. The experimental results indicate that when the enzymatic hydrolysis time is 2.5 h and 3 h, there is no significant difference in the DH and Gln content of the hydrolysate (*p* > 0.05). Therefore, to enhance enzymatic hydrolysis efficiency, 2.5 h is selected as the optimal enzymatic hydrolysis time.

Figure 2d illustrates the effect of pH during enzymatic hydrolysis on hydrolysate properties. As pH increases, the hydrolysate’s DH and Gln content first rise and then decrease. At pH 8, the hydrolysate exhibits the highest DH (12.44 ± 0.15%) and Gln content (8.68 ± 0.17%). The activity of proteases is determined by their structure. When the pH of the environment changes, the structure of proteases also changes, which may lead to a decrease in enzyme activity [27]. Trypsin is primarily found in the animal intestine, where the pH environment ranges between 7 and 8. This may explain why results are more favorable under pH 8 conditions. Based on the experimental results, pH 8 should be selected as the optimal pH for enzymatic hydrolysis.

#### 3.1.3. Response Surface Experiment

Based on the results of the single-factor experiment, the response surface methodology was employed to further optimize the enzymatic hydrolysis conditions. Enzymatic hydrolysis time (A), enzymatic hydrolysis temperature (B), and enzyme addition (C) were selected as influencing factors, with glutamine content (Y) as the response variable. A three-factor, three-level response surface experiment was designed using the Box–Behnken central composite principle, with results shown in Table 3. Performing multiple linear regression analysis on the data in Table 3 yields the regression equation for Gln content (Y):

Y = 8.75 + 0.55A + 0.12B + 0.80C − 0.038AB + 0.11AC + 0.29BC − 0.40A^2^ − 0.94B^2^ − 0.53C^2^

The results of the variance analysis for the regression model are shown in Table 4. The data indicate that the model’s F-value reached 87.32, with a *p*-value less than 0.0001, proving that the model is highly significant. The model R^2^ is 0.9912, and the lack of fit is not significant, indicating that the model adequately describes the functional relationship between the experimental factors and the response values, and the prediction of results is reliable. The *p*-values indicate that enzymatic hydrolysis time (A), enzymatic hydrolysis temperature (B), and enzyme addition (C) all significantly affect glutamine content (response variable, Y) (*p* < 0.05). Combined with the F-values, the magnitude of influence on the response variable from the three experimental factors is ranked as follows: enzyme addition (C) > enzymatic hydrolysis time (A) > enzymatic hydrolysis temperature (B). The center point replicates yielded Gln values ranging from 8.55% to 8.94%, with a mean of 8.75% and a coefficient of variation (CV) of 1.83%, confirming acceptable repeatability of the experimental system. The observed variation is attributable to the inherent complexity of the enzymatic hydrolysis process, including slight fluctuations in temperature and pH control, as well as the multi-step analytical workflow for free Gln quantification. The ANOVA results, combined with this repeatability assessment, collectively support the adequacy of the fitted model.

**Figure 3:**
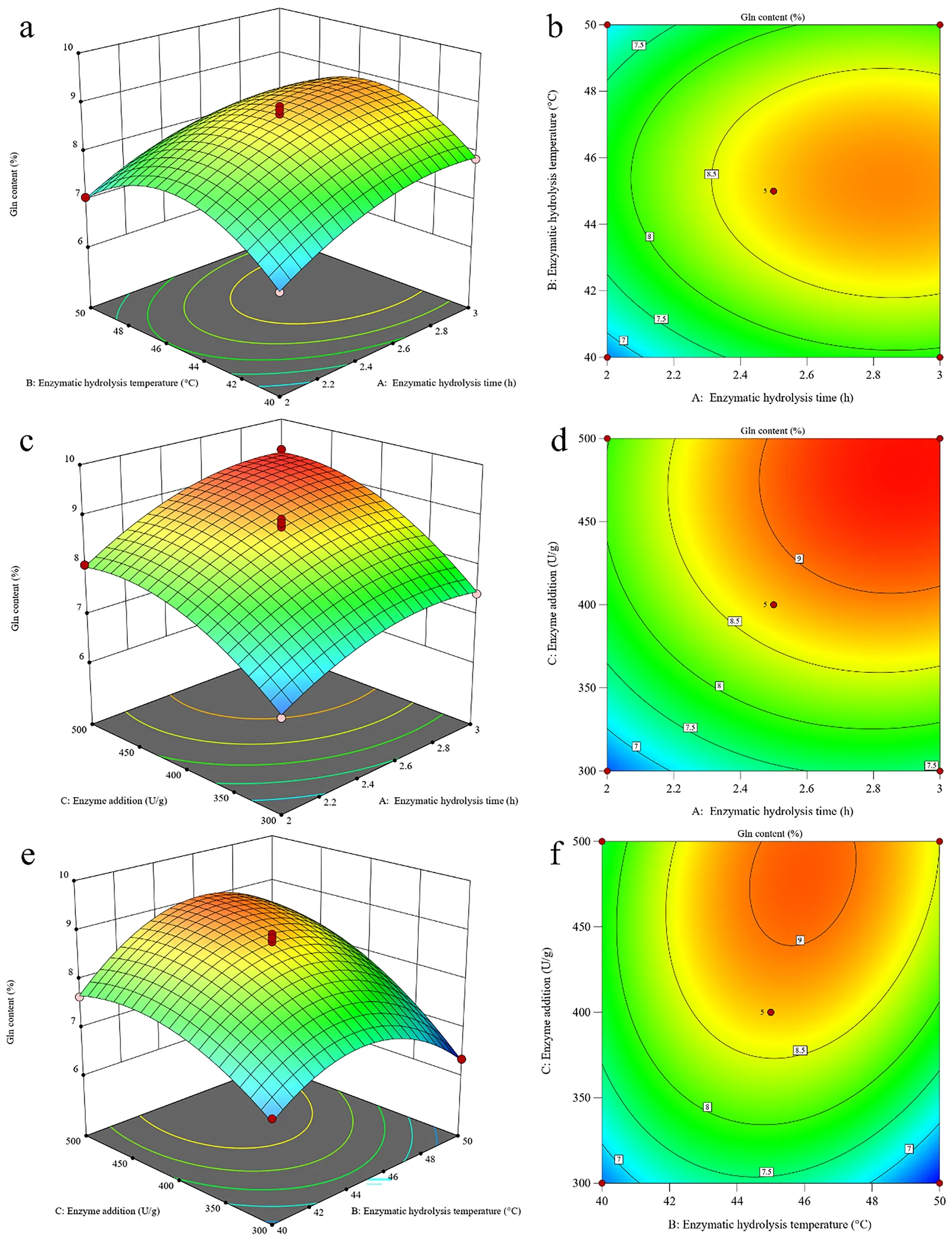
The three-dimensional response surface plot and contour plot represent the influence of the combinations of three parameters on the Gln content of the hydrolysate. (**a**,**b**) Enzymatic hydrolysis time and enzymatic hydrolysis temperature, (**c**,**d**) enzymatic hydrolysis time and Enzyme addition, (**e**,**f**) enzymatic hydrolysis temperature and enzyme addition.

Figure 3 shows the response surface plot and contour plots of the model, visually illustrating the interactions among the various factors. The response surfaces for enzymatic hydrolysis temperature (B) and enzyme addition (C) exhibit steep slopes (Figure 3e), with contour lines forming dense elliptical patterns (Figure 3f). This indicates a significant interaction between B and C, followed by A and C, and then A and B. This finding aligns with the model’s ANOVA results, which show a hierarchical interaction effect: BC > AC > AB.

The optimal enzymatic hydrolysis conditions derived from the model are as follows: enzymatic hydrolysis time of 2.9 h, enzymatic hydrolysis temperature of 45.93 °C, enzyme addition of 488.80 U/g, and theoretical Gln content of 9.34%. For practical production purposes, the enzymatic hydrolysis conditions were set as follows: enzymatic hydrolysis time of 3 h, enzymatic hydrolysis temperature of 45 °C, and enzyme addition of 480 U/g, resulting in a final Gln content of 9.43 ± 0.13%. At this point, the DH of the hydrolysate was 14.51 ± 0.16%. The model-predicted optimum Gln content was 9.34%, while the experimentally verified value under the optimized conditions was 9.43 ± 0.13%. The relative deviation of less than 1% and the overlap of the predicted value with the 95% confidence interval of the experimental mean indicate that the model provides a reliable prediction of the optimum. The slight positive deviation may reflect the fact that verification experiments, conducted under precisely controlled conditions dedicated to the predicted optimum, achieved marginally better process control than the original design runs, which were distributed across the entire experimental domain. The results indicate that the enzymatic hydrolysis conditions determined through single-factor combined response surface optimization can produce glutamine-rich corn protein hydrolysate (GRCH), offering an efficient and economical solution for utilizing Gln in corn gluten meal.

### 3.2. The Classification of GRCH and the In Vitro Antioxidant Capacity of Each Component

GRCH was fractionated using ultrafiltration technology, yielding three components with distinct molecular weights: GRCH-1 (>5 kDa), GRCH-2 (3–5 kDa), and GRCH-3 (<3 kDa). The Gln content and in vitro antioxidant activity of these fractions are shown in Table 5. Among the fractions, GRCH-2 exhibited the highest Gln content (9.08 ± 0.11%), followed by GRCH-3 (8.64 ± 0.15%), indicating that most Gln was enriched in fractions with molecular weights below 5 kDa.

Preliminary in vitro evaluations of the antioxidant capacity of each component revealed that all components exhibited certain hydroxyl radical scavenging capacity, ABTS radical scavenging capacity, and Fe^2+^ chelation capacity, all of which were higher than that of GRCH. Among these components, GRCH-3 exhibited the highest radical scavenging capacity and relatively high Fe^2+^ chelation ability. This finding is consistent with previous studies indicating that components with lower molecular weights often possess higher activity [28]. Glutamine peptides demonstrate significant advantages in maintaining intestinal health, largely due to their antioxidant capacity. Certain glutamine peptides can scavenge free radicals within intestinal tissues, inhibit lipid peroxidation, and regulate the expression of antioxidant enzyme systems [4]. GRCH and its individual components demonstrated antioxidant activity in vitro, potentially exerting beneficial effects on oxidative homeostasis in the gut. Consequently, GRCH-3, which exhibits superior antioxidant capacity and relatively higher Gln content, was selected for subsequent analysis.

### 3.3. Identification of Glutamine Peptides and Analysis of Physicochemical Parameters

To further elucidate the structure–activity relationship of glutamine peptides, the GRCH-3 fraction was further separated using gel chromatography, and fractions with molecular weights less than 1 kDa were intercepted. The peptide sequences of this fraction were identified by Nano LC-MS/MS, resulting in 1479 peptide sequences. Peptide relative abundance was determined based on extracted ion chromatogram (XIC) peak intensities. For each identified peptide, the XIC peak area was integrated and normalized to the total ion intensity of all detected peptides in the same run. Peptides with a relative intensity ≥0.01% were considered for further analysis. This threshold was applied to filter out low-abundance signals that may represent analytical noise or minor degradation products. Based on the abundance ratio, select peptide sequences with a content above 0.01% (559 peptide sequences) for physicochemical parameter analysis, especially peptide sequences containing Q (175 peptide sequences). Provide data support for the subsequent screening of glutamine peptides with potential biological activity. The physicochemical parameters analysis results of 175 Q-containing peptide sequences are presented in Table S1.

**Figure 4:**
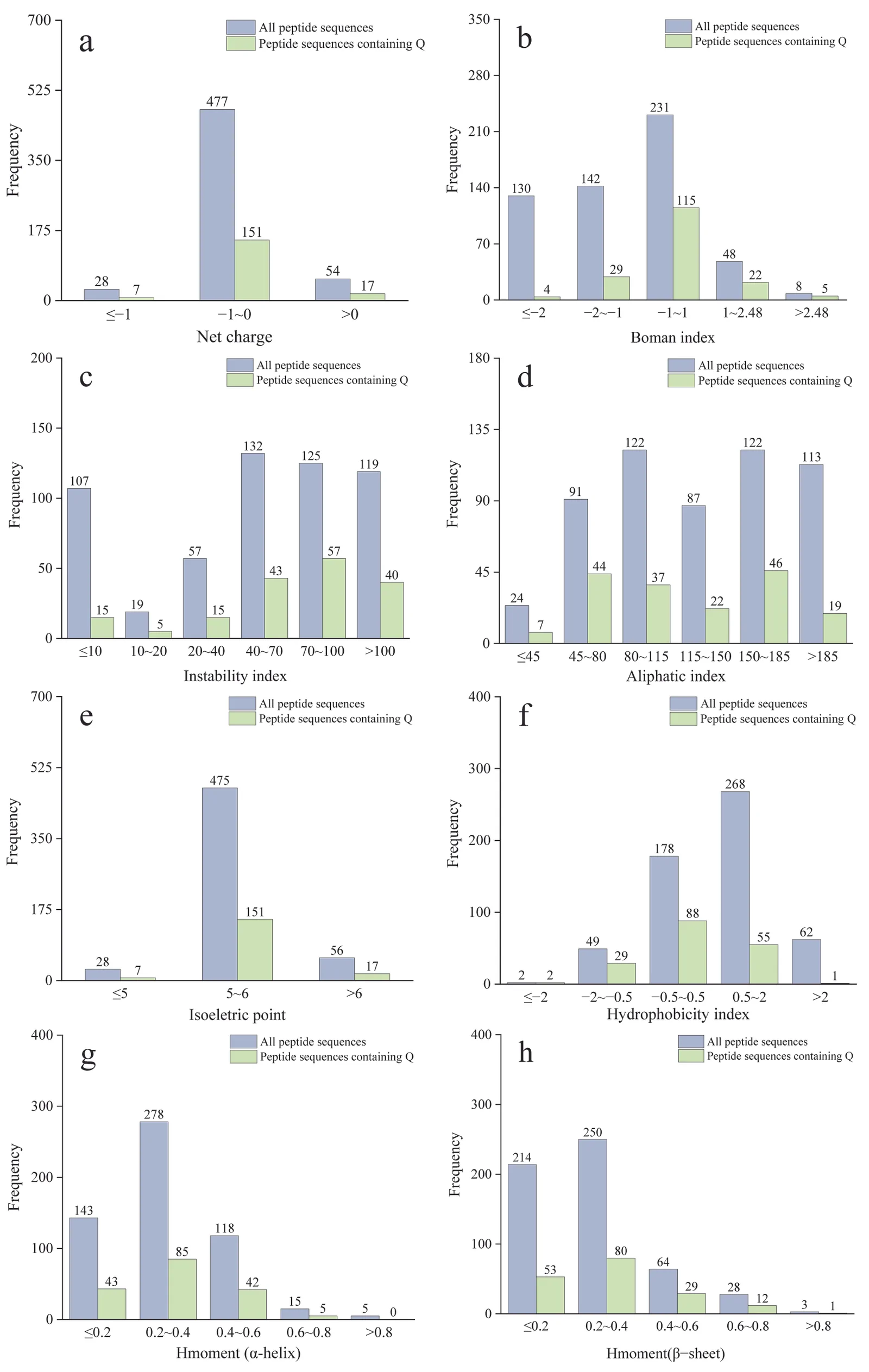
The physicochemical parameters of the identified peptide sequences that account for the top 0.01% of relative abundance. (**a**) Net charge, (**b**) Boman index, (**c**) Instability index, (**d**) Aliphatic index, (**e**) Isoelectric point, (**f**) Hydrophobicity, (**g**) Hmoment α-helix, (**h**) Hmoment β-sheet.

Figure 4a shows the net charge analysis results for the aforementioned peptide sequences. At pH 7, the net charge carried by most peptides approaches zero. Among the 54 peptides with positive net charge, basic amino acid residues (such as Lys, Arg, His) appear frequently, while the 28 peptides with net charge ≤ −1 are rich in acidic amino acid residues (such as Asp, Glu). The presence of these amino acid residues determines the overall charge properties of the peptides. The net charge distribution trend of Q-containing peptide sequences aligns with that of the overall peptide sequence, with most peptides exhibiting net charges ranging from 0 to −1. This indicates that the majority of glutamine peptides maintain a relatively stable charge state.

The Boman index is a measure used to describe the binding potential of peptides to proteins. Higher values are generally associated with stronger predicted binding propensity and potential multifunctionality. A reference value of 2.48 was adopted from previous work as an indicator of favorable binding potential [29]. As shown in Figure 4b, the Boman index for the majority of the identified peptides, including glutamine-containing sequences, fell below 2.48, with most values distributed between −1 and 1. Eight peptide sequences exhibited a Boman index above 2.48, five of which contained glutamine. Rather than serving as an exclusion cutoff, the 2.48 threshold was used to flag these eight peptides as high-priority candidates for subsequent molecular docking analysis. The final selection of lead peptides was determined through an integrated multi-parameter evaluation, combining Boman index ranking, bioactivity prediction scores, and molecular docking results, thereby avoiding over-reliance on any single in silico parameter.

The Instability Index is a metric for predicting peptide stability, calculated based on the peptide’s primary structure. Peptides with an Instability Index below 40 are predicted to be stable [30]. Figure 4c shows the instability index for the identified peptide sequences. Among these, 183 peptide sequences had an index below 40, accounting for 32.74% of the total. This indicates that the majority of peptides are likely unstable. There are 35 Q-containing peptide sequences with an instability index below 40, which can be considered stable.

The aliphatic index serves as a measure of thermal stability, calculated based on the relative volume of the side chains of aliphatic amino acids (Ala, Ile, Leu, and Val). A higher aliphatic index indicates greater thermal stability [31]. Figure 4d shows the aliphatic index of the identified peptide sequences, ranging from 0 to 312 with an average value of 134.8. Most peptide sequences are distributed within the higher aliphatic index range. The aliphatic index distribution of Q-containing peptide sequences ranges from 0 to 292.5, with an average value of 122. Overall, the aliphatic index of Q-containing peptide sequences is lower compared to the total peptide sequences, likely due to the slightly reduced content of aliphatic amino acid residues in most Q-containing peptide sequences.

Figure 4e shows the isoelectric point (pI) calculation results for the identified peptide sequences. Overall, the pI distribution trend of the peptide sequences is similar to the net charge distribution trend. The pI values of the vast majority of peptide sequences (including Q-containing peptide sequences) are concentrated between 5 and 6. Notably, 465 peptide sequences (including 148 Q-containing peptide sequences) have a pI of 5.5. This suggests that most peptides may exhibit good solubility in neutral environments. However, the impact of peptide hydrophobicity in practical applications still requires consideration.

Figure 4f shows the hydrophobicity index analysis results for the identified peptide sequences. Peptides with a hydrophobicity index between −0.5 and 0.5 are defined as amphiphilic peptides, while those with indices below −0.5 and above 0.5 are defined as hydrophilic and hydrophobic peptides, respectively [32]. Among all identified peptide sequences, amphiphilic peptides, hydrophilic peptides, and hydrophobic peptides numbered 178, 51, and 330, respectively. Within Q-containing peptide sequences, these three types numbered 88, 31, and 56, respectively. Overall, Q-containing peptides appear more hydrophilic, potentially due to the polar side chain of Gln.

The hydrophobic moment serves as a measure of peptide amphiphilicity, predicting the likelihood of a peptide forming amphiphilic structures such as α-helix or β-sheet. Such amphiphilic structures may contribute to the peptide’s transmembrane properties [33,34]. As shown in Figure 4g,h, Hmoment (α-helix) and Hmoment (β-sheet) represent the predicted values for the formation of the α-helix and β-sheet structures in the identified peptide sequence, respectively. The predicted values for most peptide sequences are below 0.4. Among these, 75% of peptide sequences with Hmoment (α-helix) below 0.4 and 83% with Hmoment (β-sheet) below 0.4 account for the total. Overall, it appears that the likelihood of forming an α-helix structure is higher. For Q-containing peptide sequences, the probabilities of forming the two structures are comparable. The proportion with Hmoment (α-helix) below 0.4 is 73%, while that with Hmoment (β-sheet) below 0.4 is 76%.

### 3.4. Computer-Aided Glutamine Peptide Screening and Activity Prediction

#### 3.4.1. Screening Based on Physicochemical Parameters

The ultimate efficacy of bioactive peptides depends on their ability to retain activity upon reaching their target sites. Therefore, this study sought to identify stable and effective glutamine peptides by analyzing physicochemical parameters of identified peptide sequences, focusing on aspects such as peptide activity, stability, and transmembrane absorption.

Low molecular weight is not only typically associated with higher biological activity but also maintains greater stability during digestion due to its simpler structure and fewer enzymatic cleavage sites [35]. The molecular weights of peptides identified by this institute are all below 1 kDa, a characteristic that lays a solid foundation for subsequent screening of glutamine peptides with potential biological activity. The instability index and aliphatic index are metrics reflecting peptide stability. During screening, sequences with an instability index below 40 and the highest possible aliphatic index should be prioritized. The Boman index is one of the key parameters considered, with a high Boman index indicating the peptide’s high potential for activity. The distribution of amino acids is also a factor to consider. Hydrophobic amino acid content and distribution were also considered, as consecutive hydrophobic residues can form stable hydrophobic patches that facilitate peptide–protein interactions [36]. Regarding peptide absorption, both amphiphilic peptides and high hydrophobic moment scores were considered. Based on the above principles, five glutamine peptides HLLGQ, QYPL, RQPQCSPL, SFQQS, and QFSLP were selected for subsequent studies. The physicochemical parameters of these peptides meet most of the aforementioned requirements. Furthermore, predictions from the ToxinPred server indicate that none of these peptides exhibit potential toxicity (Table 6).

#### 3.4.2. Activity Prediction Based on Molecular Docking

Glutamine peptides not only provide Gln as a nutritional supplement but also play a role in regulating biological signaling pathways, which is significant for improving intestinal inflammation [4]. A study on the targets of food-derived peptides in colitis showed that the inflammatory bowel cancer-related signaling pathway JAK2/STAT3 was the key pathway for peptides involved in inflammation regulation, and these peptides mainly interacted with the core targets JAK2 and STAT3 through hydrogen bonding [21]. To investigate the potential effects of the aforementioned five glutamine peptides on intestinal inflammation, molecular docking analysis was performed for each peptide with JAK2 and STAT3, enabling visualization of interactions between the peptides and their target proteins. The results are presented in Table 6 and Table 7.

**Figure 5:**
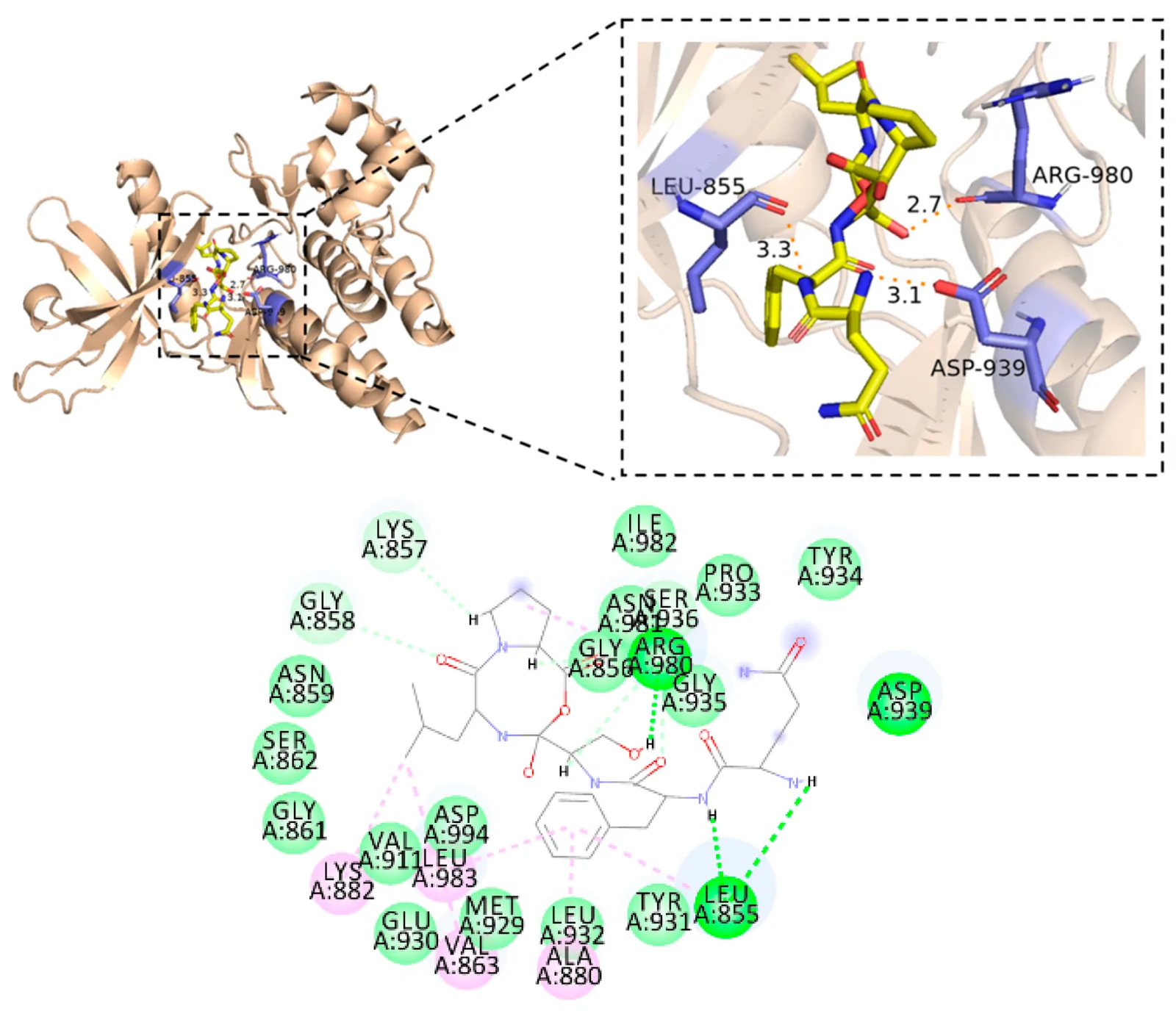
Molecular docking diagram of QFSLP and JAK2. Light green represents van der Waals, dark green represents hydrogen bonds, and pink represents Pi-Alkyl.

**Figure 6:**
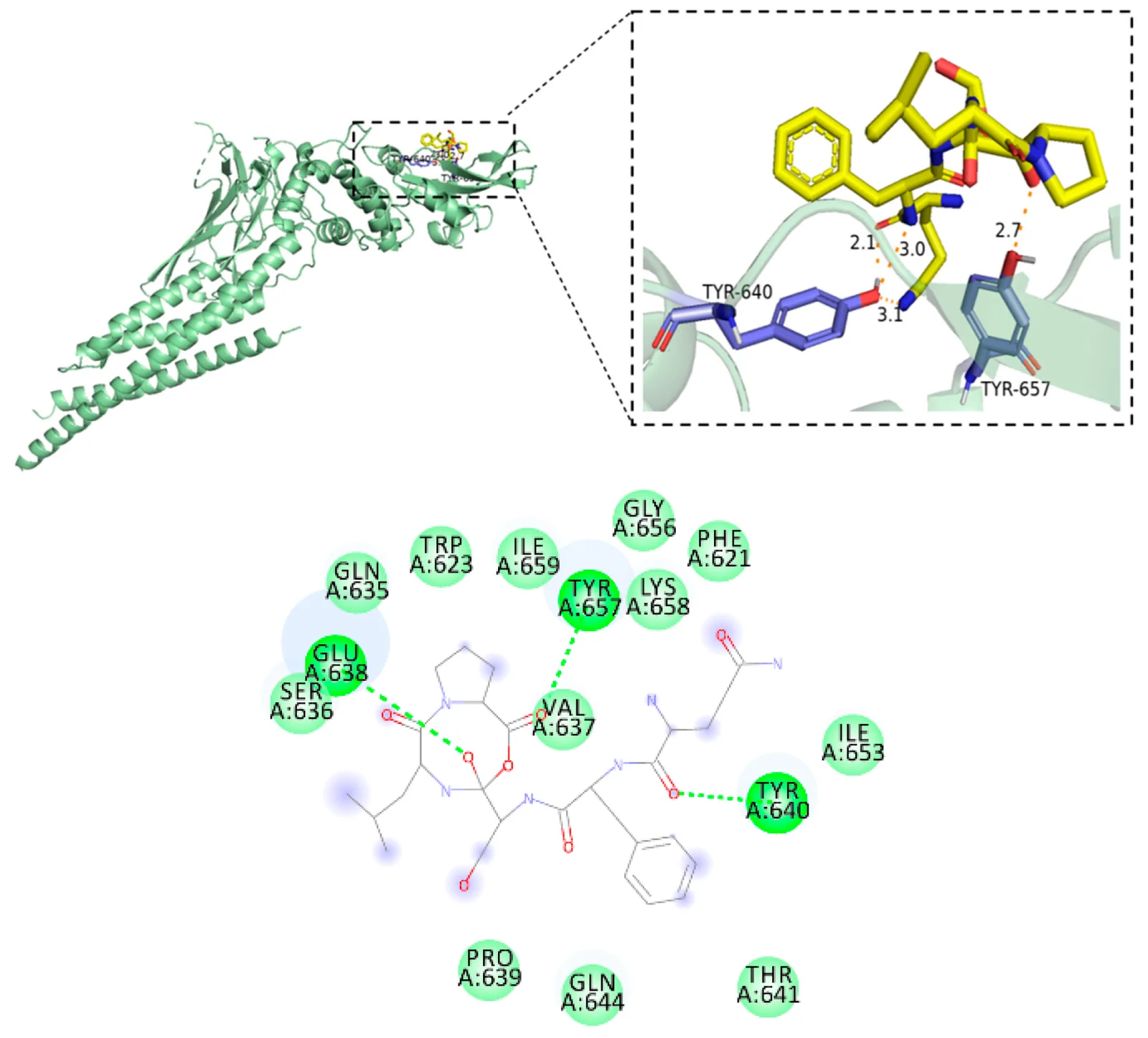
Molecular docking diagram of QFSLP and STAT3. Light green represents van der Waals, and dark green represents hydrogen bonds.

Molecular docking results indicate that all five glutamine peptides establish stable interactions with JAK2 and STAT3, with peptide–protein binding energies exceeding −5 kcal/mol, with the binding energies between peptides and JAK2 ranging from −8.0 to −8.9 kcal/mol, and those between peptides and STAT3 ranging from −6.0 to −7.3 kcal/mol. The binding of peptides to proteins is driven by both hydrophobic effects and hydrogen bonds. Among them, the hydrophobic effect mainly relies on van der Waals forces, while part of the van der Waals forces are provided by the Pi-Alkyl interaction. QFSLP is the peptide with the strongest binding affinity for JAK2 and STAT3, exhibiting binding energies of −8.9 kcal/mol and −7.3 kcal/mol, respectively. Its interactions with JAK2 and STAT3 are illustrated in Figure 5 and Figure 6.

The JAK2/STAT3 signaling pathway is a classical phosphorylation cascade. When a cytokine binds to its receptor on the cell membrane, it triggers the dimerization of the receptor and activates the conjugated JAK2, resulting in the phosphorylation of JAK2 to form a STAT3 binding site in the cytoplasm. STAT3 recruited to the site is phosphorylated to form a dimer, which then dissociates and migrates to the nucleus and finally integrates into a specific DNA sequence to regulate gene transcription [37]. In the progression of intestinal inflammation, activation of the JAK2/STAT3 signaling pathway exacerbates inflammation, damages the intestinal barrier, and induces apoptosis in intestinal epithelial cells [38]. Inhibiting the abnormal activation of the JAK2/STAT3 signaling pathway is an effective strategy for reducing intestinal inflammatory responses. CYT387 is an ATP-competitive small molecule that significantly inhibits JAK2 at low concentrations [39]. Once CYT387 occupies the ATP-binding pocket of JAK2, JAK2 is unable to obtain phosphate from ATP to complete phosphorylation, thereby completely blocking the signaling pathway. Studies indicate that CYT387 achieves stable binding with the key residues Leu932, Lys943, Leu855, Val911, Leu983, Tyr934, Ala880, and Val863 of JAK2 [40]. 323-2 is a STAT3 inhibitor that directly targets the SH2 domain of STAT3, which mediates STAT3 phosphorylation and dimerization. The 323-2 forms bonds with the key residues Gln635, Glu638, Ile634, Ser636, Val637, Arg595, and Lys591 in the STAT3 SH2 domain [41]. Notably, the top-ranked binding poses of QFSLP from blind docking were found to localize within or adjacent to regions corresponding to the known inhibitor-binding or ATP-binding pockets of JAK2 and STAT3, suggesting a potential for functional interference. In this study, five glutamine peptides exhibited partial overlap in their binding sites on the target protein with the aforementioned JAK2/STAT3 inhibitors (Table 7 and Table 8). This suggests that these peptides may bind to the same or similar locations on the target protein as the inhibitors, indicating they may exert comparable effects. QFSLP is the most promising glutamine peptide for inhibiting the JAK2/STAT3 pathway. Not only does it possess the highest binding energy score, but its binding site on JAK2 also highly overlaps with that of CYT387 (7 residues), differing by only one residue position. Additionally, the molecular docking results presented here are based on single energy-minimized conformers of each peptide. Short linear peptides are known to sample a broad conformational space in solution, and the use of a single conformer may not capture the full range of binding-competent geometries. Future computational studies employing specialized peptide structure prediction tools (e.g., PEP-FOLD or Rosetta) to generate conformational ensembles, coupled with ensemble docking or molecular dynamics simulations, will be necessary to more reliably characterize the interactions between these glutamine peptides and JAK2/STAT3.

### 3.5. The Transport of QFSLP in Caco-2 Cell Monolayer

Based on preliminary research findings, QFSLP is believed to potentially inhibit the JAK2/STAT3 signaling pathway, which may alleviate intestinal inflammation. However, the effective exertion of this activity depends on whether the peptide can be fully absorbed by intestinal epithelial cells. To validate this critical premise, this study employed a human colon adenocarcinoma cell (Caco-2) monolayer model to simulate the intestinal epithelial barrier and examined the transport of QFSLP obtained via solid-phase synthesis in this model.

**Figure 7:**
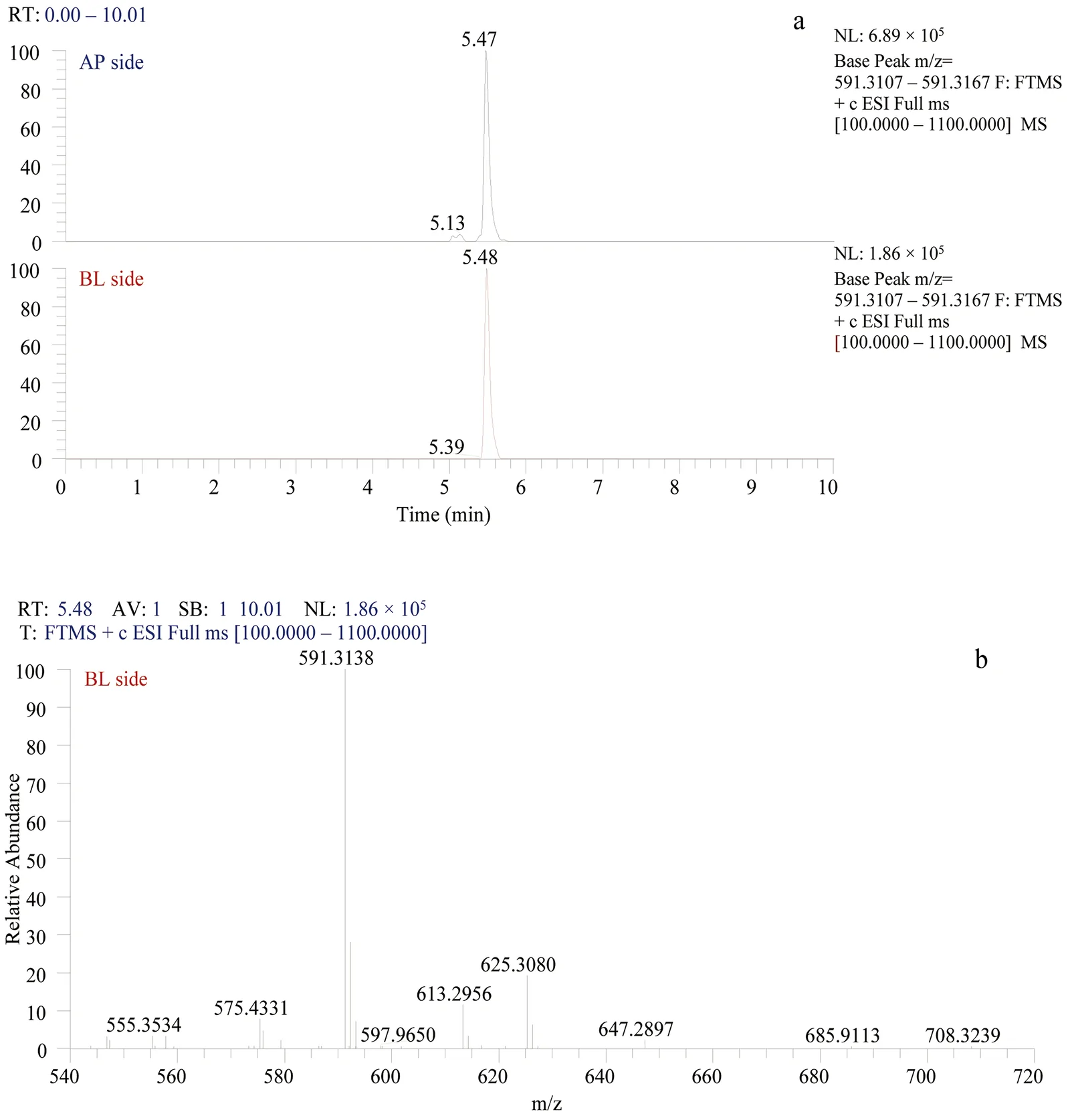
The mass spectra of QFSLP on the AP side and BL side of the Caco-2 cell monolayer. (**a**) The EICs of QFSLP on the AP side and BL side. (**b**) The MS1 of QFSLP on the BL side.

The presence of QFSLP in the BL side of the Caco-2 cell monolayer was detected using LC-MS, with results shown in Figure 6. By extracting the total ion chromatogram from the AP and BL sides at the *m*/*z* of QFSLP ([M + H^+^]), the corresponding extracted ion chromatogram (EIC) was obtained. As shown in the figure, both EICs exhibit distinct peak signals at approximately 5.48 min (AP side: 5.47 min; BL side: 5.48 min), with highly consistent retention times (Figure 7a). Further analysis of the peak at 5.48 min on the BL side revealed that its primary mass spectrum (MS1) showed a base peak *m*/*z* of 591.3138 (Figure 7b), which closely matched the *m*/*z* of the target peptide QFSLP ([M + H^+^]). This indicates the presence of intact QFSLP on the BL side.

The primary pathways for peptide absorption by intestinal epithelial cells are the transcellular pathway and the paracellular pathway. The transcellular pathway, based on specific mechanisms, further includes carrier-mediated active transport, passive diffusion, and endocytosis [42]. Certain amphipathic peptides with transmembrane capabilities tend to form amphipathic structures within membrane environments. These structures comprise two planes, one hydrophilic and one hydrophobic, allowing the hydrophobic surface to embed within the membrane’s hydrophobic core while the hydrophilic surface faces the hydrophilic region. This induces membrane deformation or inversion, thereby facilitating peptide internalization [43]. The physicochemical parameter analysis in this study indicates that QFSLP is an amphiphilic peptide with a high hydrophobic moment, suggesting a strong tendency to form amphiphilic structures in membrane environments. It may be absorbed by the intestinal epithelium via transcellular pathways. However, the exact absorption mechanism of it in the Caco-2 cell monolayer model, including the specific internalization pathway and whether it involves the paracellular pathway, awaits further experimental clarification.

### 3.6. Effect of QFSLP on LPS-Induced Inflammation in Caco-2 Cells

**Figure 8:**
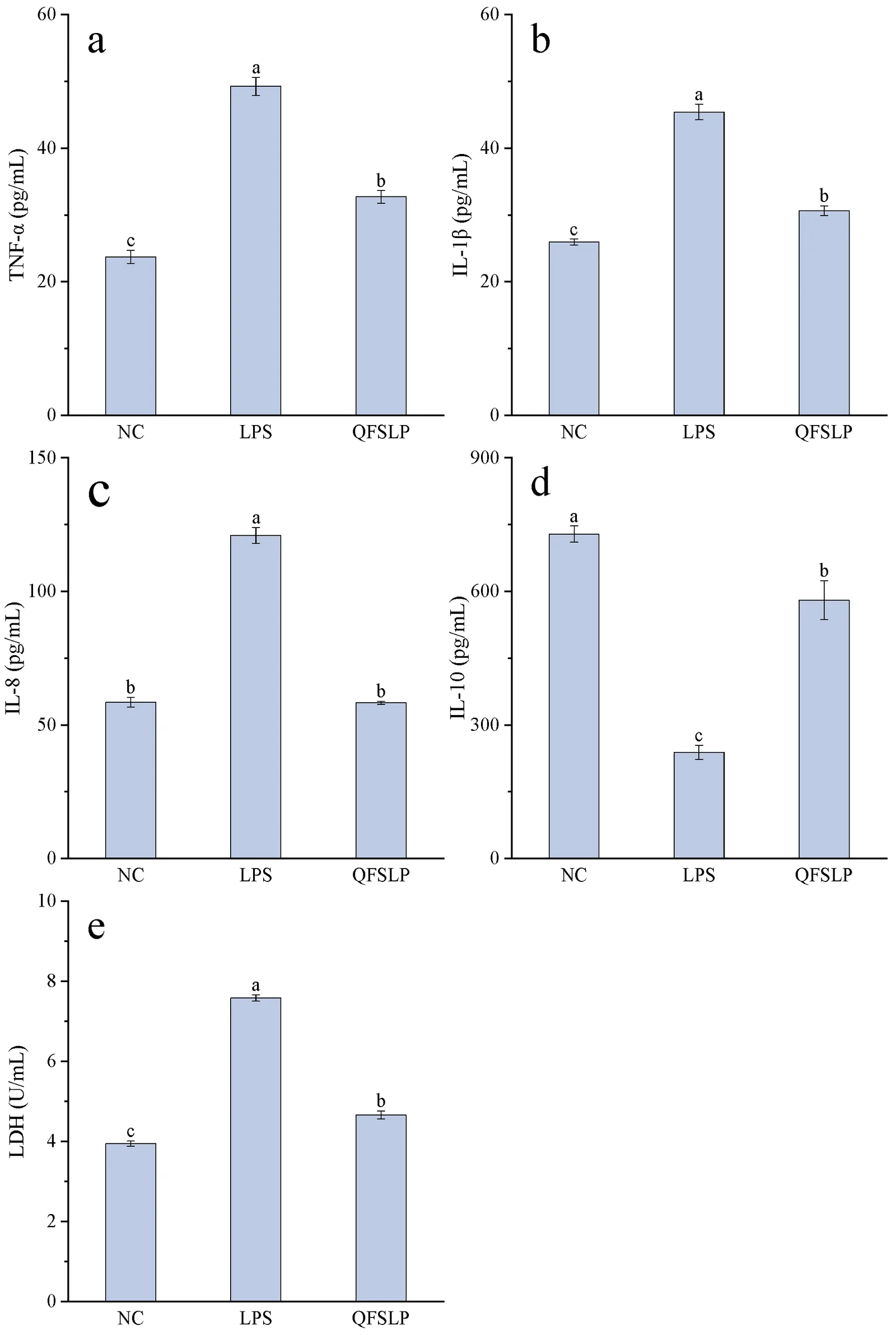
Effect of QFSLP on the levels of inflammatory markers in the inflammatory model of Caco-2 cells induced by LPS (**a**) TNF-α, (**b**) IL-1β, (**c**) IL-8, (**d**) IL-10, (**e**) LDH. Different lowercase letters represent indicate significantly different (*p* < 0.05).

The Caco-2 cell line is derived from human colorectal adenocarcinoma. Due to their ability to express the morphological and functional characteristics of intestinal epithelial cells after differentiation, Caco-2 cells have been established as a classic intestinal epithelial model. Furthermore, this cell line has been demonstrated to respond to external stimuli, such as lipopolysaccharide (LPS), on microbial membranes, thereby secreting inflammatory markers. Based on this characteristic, the Caco-2 cell model is frequently used to study the absorption and transport of bioactive compounds, as well as their effects on intestinal inflammation [44]. This study established an LPS-induced inflammatory model in Caco-2 cells and further investigated the effects of QFSLP intervention on inflammatory marker levels, with results shown in Figure 8.

Compared to normal cells, cells in the LPS-stimulated model group exhibited a strong inflammatory response. The model group exhibited significantly elevated secretion levels of proinflammatory cytokines TNF-α, IL-1β, and IL-8 (*p* < 0.05), increasing by 107.8%, 75.1%, and 106.8%, respectively. In contrast, the content of the anti-inflammatory cytokine IL-10 was significantly reduced (*p* < 0.05), decreasing by approximately 67.3%. Additionally, LDH activity in the cell culture supernatant of the model group increased by 92.4%, significantly higher than that in normal cells (*p* < 0.05). This indicates LDH leakage from cells, suggesting that the inflammatory response may have caused cellular damage.

Compared with the model group, intervention with QFSLP (100 μg/mL) effectively alleviated inflammatory responses in Caco-2 cells. TNF-α and IL-1β levels in the intervention group increased by only approximately 37.9% and 18.2%, respectively, compared with normal cells, while IL-8 levels showed no significant difference (*p* > 0.05). Compared with the control group, the QFSLP intervention significantly increased the secretion level of the anti-inflammatory factor IL-10 (*p* < 0.05), with a 143.2% increase. Concurrently, LDH activity in the intervention group’s cell supernatants was only 18.3% higher than that in normal cells, indicating that inflammation-related cellular damage was markedly suppressed.

The present results demonstrate that QFSLP effectively mitigated the LPS-induced inflammatory cascade in Caco-2 cells. LPS triggered a typical inflammatory response characterized by robust upregulation of TNF-α, IL-1β, and IL-8, concomitant with a sharp decline in the anti-inflammatory cytokine IL-10. This imbalance mirrors the cytokine profile observed in intestinal inflammation, where overproduction of proinflammatory mediators coupled with insufficient anti-inflammatory feedback contributes to epithelial injury. The parallel increase in LDH release further confirmed the loss of membrane integrity, likely as a consequence of both direct cytokine-mediated cytotoxicity and secondary oxidative stress.

IL-10 is an important anti-inflammatory factor within cells. As can be observed from Figure 8d, compared with the control group, after being treated with LPS, the content of IL-10 in the cells significantly decreased. After the QFSLP intervention, the content of IL-10 in the cells significantly increased, indicating that the intervention of the peptide can enhance the cells’ resistance to inflammation. The notable 143.2% increase in IL-10 secretion following QFSLP intervention warrants further consideration, given the central role of IL-10 in resolving intestinal inflammation and restoring mucosal homeostasis. IL-10 expression is canonically regulated by the JAK/STAT signaling cascade, particularly through STAT3-mediated transcriptional activation. Whether QFSLP directly engages this pathway remains to be determined. Given that QFSLP was initially identified through molecular docking screening against JAK2 and STAT3, it is plausible that the observed IL-10 elevation reflects, at least in part, the modulation of this signaling axis. However, in the absence of direct protein-level evidence, this interpretation remains a working hypothesis, and alternative mechanisms—such as relief of LPS-mediated transcriptional repression or indirect effects through redox modulation—cannot be excluded. If validated, this mechanism would represent a meaningful upstream signaling complement to the well-documented NF-κB-inhibitory effects of glutamine, suggesting that QFSLP could orchestrate a coordinated anti-inflammatory response by simultaneously suppressing proinflammatory NF-κB signaling and potentiating the anti-inflammatory JAK2/STAT3/IL-10 axis.

Consistent with the attenuation of cytokine dysregulation, QFSLP dramatically reduced LDH leakage, implying preservation of plasma membrane integrity. This observation aligns with previous reports that glutamine supplementation supports intestinal barrier function by upregulating tight junction proteins such as occludin and claudin-1 [7]. Whether QFSLP exerts its cytoprotective effect solely through glutamine availability or also via peptide-specific signalling remains an open question. It is plausible that the QFSLP sequence possesses intrinsic bioactivity beyond serving as a glutamine carrier, particularly if the peptide interacts with cell surface receptors or is internalised intact.

The present study monitored endpoints only at the cytokine and LDH level; direct evidence for barrier function and tight junction protein expression is lacking. Furthermore, the activation status of NF-κB, MAPK, and STAT3 was not assessed at the protein level in this study. Consequently, the proposed mechanistic model, in which QFSLP attenuates NF-κB-driven inflammation while potentially engaging STAT3-mediated IL-10 production, should be regarded as a framework for future investigation rather than an established pathway. Direct evidence for the involvement of these signalling nodes will require phospho-specific protein analysis and pathway-specific inhibitor or knockdown approaches to substantiate the preventive potential of QFSLP against intestinal injury.

## 4. Conclusions

This study established an efficient enzymatic hydrolysis system capable of releasing glutamine peptides from corn gluten meal, yielding glutamine-rich corn protein hydrolysate GRCH. Among these, the fraction GRCH-3 with a molecular weight below 3 kDa exhibits certain in vitro antioxidant capacity. From this fraction, 175 Q-containing peptide sequences with molecular weights below 1 kDa were identified. Through physicochemical parameter analysis and functional prediction via molecular docking, five glutamine peptides with potential inhibitory effects on the JAK2/STAT3 signalling pathway were identified, among which QFSLP demonstrated the most promising potential. In vitro experiments indicate that QFSLP can be fully transported across Caco-2 cell monolayers and significantly alleviates LPS-induced inflammation in Caco-2 cells. This study provides a theoretical basis for the efficient utilisation of glutamine resources in corn gluten meal. It also offers data supporting the efficacy of glutamine peptide QFSLP in alleviating intestinal inflammation, thereby establishing a highly promising foundational ingredient for the development of functional foods related to gut health. Investigating how QFSLP antagonises inflammation via the JAK2/STAT3 signalling pathway and elucidating its absorption mechanism will be the primary focus of our subsequent research.

## Figures and Tables

**Table 1:** Enzymatic conditions of different proteases.

Proteases	Substrate Concentration (%)	Enzyme Addition (U/g)	Temperature (°C)	Time (h)	pH
Protamex	10	1300	55	2.5	7.0
Neutrase	10	600	45	2	7.0
Flavourzyme	10	600	50	2	6.5
Papain	10	600	50	2	6.5
Trypsin	10	600	40	2	7.5

**Table 2:** Response surface test levels and factor design.

Levels	Factors		
	A (Time, h)	B (Temperature, °C)	C (Enzyme Addition, U/g)
−1	2	40	300
0	2.5	45	400
1	3	50	500

**Table 3:** Response surface test protocol and results.

Run	A Time (h)	B Temperature (°C)	C Enzyme Addition (U/g)	Gln Content (%)
1	2.5	45	400	8.78
2	2	40	400	6.71
3	2.5	50	500	8.43
4	2.5	45	400	8.64
5	2.5	45	400	8.86
6	2.5	45	400	8.94
7	2	50	400	7.06
8	3	45	300	7.43
9	2.5	40	300	6.74
10	2	45	300	6.53
11	2	45	500	8.02
12	3	50	400	8.06
13	3	45	500	9.34
14	2.5	50	300	6.34
15	2.5	40	500	7.65
16	3	40	400	7.86
17	2.5	45	400	8.55

**Table 4:** Analysis of variance for regression model.

Source	Sum of Squares	DF	Mean Square	F-Value	*p*-Value	
Model	14.07	9	1.56	87.32	<0.0001	significant
A	2.39	1	2.39	133.31	<0.0001	
B	0.1081	1	0.1081	6.04	0.0436	
C	5.12	1	5.12	285.93	<0.0001	
AB	0.0056	1	0.0056	0.3141	0.5926	
AC	0.0441	1	0.0441	2.46	0.1606	
BC	0.3481	1	0.3481	19.44	0.0031	
A^2^	0.6594	1	0.6594	36.83	0.0005	
B^2^	3.69	1	3.69	205.9	<0.0001	
C^2^	1.17	1	1.17	65.62	<0.0001	
Residual	0.1253	7	0.0179			
Lack of Fit	0.0242	3	0.0081	0.3194	0.8122	not significant
Pure Error	0.1011	4	0.0253			
Cor Total	14.20	16				
R^2^ = 0.9912	AdjR^2^ = 0.9798					

**Table 5:** Gln content, free radical scavenging capacity and Fe^2+^ chelating ability of ultrafiltration fractions.

Fractions	Gln Content (%)	IC_50_ (mg/mL)		
		Hydroxyl	ABTS	Fe^2+^
GRCH	9.43 ± 0.13 a	2.14 a	0.012 b	2.05 a
GRCH-1	3.40 ± 0.18 c	1.47 b	0.042 a	1.01 b
GRCH-2	9.08 ± 0.11 a	0.98 c	0.045 a	1.33 b
GRCH-3	8.64 ± 0.15 b	0.77 c	0.004 c	1.16 b

Different lowercase letters represent significant difference (*p* < 0.05).

**Table 6:** Physicochemical properties of the preselected peptide sequences.

Sequence of Peptide	Net Charge	Boman Index	Instability Index	Aliphatic Index	Isoelectric Point	Hydrophobicity	Hmoment (α-Helix)	Hmoment (β-Sheet)	Toxicity Prediction
HLLGQ	0.08	−0.116	8.0	156.0	6.74	0.10	0.402	0.269	Non-Toxin
QYPL	0	0.190	19.5	97.5	5.52	−0.65	0.212	0.448	Non-Toxin
RQPQCSPL	0.95	2.931	126.6	48.7	8.24	−1.15	0.213	0.135	Non-Toxin
SFQQS	0	2.980	134.4	0	5.52	−0.16	0.417	0.321	Non-Toxin
QFSLP	0	0.208	31.4	78.0	5.52	0.14	0.094	0.586	Non-Toxin

**Table 7:** The molecular docking results of 5 glutamine peptides with JAK2.

Sequence	Affinity (kcal mol^−1^)	Binding Site		
		Hydrogen Bond	Van der Waals	Pi-Alkyl
HLLGQ	−8.0	Gly858 Asp994 Asp976 Asn981 Arg980 Ser936 Leu855 Asp939	Asn859 Gly861 Lys882 Ser862 Lys857 Gly993 Val911 Leu932 Gly935 Pro933 Tyr934 Gly856 Glu1015	Val863 Met929 Ala880 Leu983
QYPL	−8.2	Arg938 Ser936 Arg980	Lys857 Ser862 Gly858 Gly861 Lys882 Asn859 Asn981 Gly935 Asp994 Glu930 Gly993 Met929	Val863 Leu932 Ala880 Leu855 Leu983 Tyr931
RQPQCSPL	−8.0	Gly993 Val863 Gly858 Lys857 Leu932 Ser936 Leu855 Gln853	Met929 Val911 Ala880 Asp994 Lys882 Gly861 Ser862 Arg980 Gly935 Gly856 Tyr934 Gln854 Tyr931 Asp939 Pro933 Arg938 Leu983 Asn981 Asn59	Lys943
SFQQS	−8.3	Leu855 Ser936 Arg980 Asp939 Gly993 Asp976 Asp994	Gly856 Gly993 Gly858 Lys857 Asn981 Asn859 Lys882 Met929 Leu932 Val911 Glu930 Tyr931 Glu1015	Ala880 Val863 Leu983
QFSLP	−8.9	Arg980 Leu855 Asp939	Gly861 Ser862 Asn859 Gly858 Lys857 Gly856 Asn981 Ile982 Ser936 Gly935 Pro933 Tyr934 Tyr931 Leu932 Met929 Glu930 Asp994 Val911	Lys880 Val863 Leu983 Ala880

Note: Red indicates overlapping binding sites of the peptides and inhibitor CYT387 on JAK2.

**Table 8:** The molecular docking results of 5 glutamine peptides with STAT3.

Sequence	Affinity (kcal mol^−1^)	Binding Site		
		Hydrogen Bond	Van der Waals	Pi-Alkyl
HLLGQ	−6.0	Lys591 Glu612 Lys557 Ser613	Val637 Arg595 Ile634 Gln635 Thr622 Glu594 Ile589 Ser590 Arg609 Ser611 Ser614 Glu638 Ser636 Thr620	Pro639
QYPL	−6.3	Tyr640 Lys658 Gln644 Met 660	Gly656 Tyr657 Val637 Glu638 Pro639 Ile659 Leu666	Met648 Ile653
RQPQCSPL	−6.0	Glu612 Arg609 Ser613 Glu638 Val637 Ser636 Glu594	Ser611 Ser614 Thr620 Pro639 Tyr623 Tyr657 Arg595 Thr622 Gln635 Ile634 Lys591	
SFQQS	−6.1	Ser636 Ile634 Glu612 Ser613 Arg609	Glu638 Gln635 Thr622 Arg595 Thr620 Pro639 Glu594 Ser614 Ser611 Lys591 Ile589 Ser590	
QFSLP	−7.3	Glu638 Tyr657 Tyr640	Ser636 Gln635 Trp623 Ile659 Val637 Gly656 Lys658 Phe621 Ile653 Thr641 Gln644 Pro639	

Note: Red indicates overlapping binding sites of the peptides and inhibitor 323-2 on STAT3.

## Data Availability

The original contributions presented in this study are included in the article/Supplementary Material. Further inquiries can be directed to the corresponding authors.

## Supplementary Materials

The following supporting information can be downloaded at https://www.mdpi.com/article/10.3390/foods15162904/s1: Table S1. Analysis results of physicochemical parameters for 175 Q-containing peptide sequences. Figure S1. The HPLC chromatogram and mass spectrum of the synthetic QFSLP peptide.
