# Supplementary material for: From Corn Gluten Meal to Bioactive Glutamine Peptides: Stepwise Enzymatic Release, Peptidomics Analysis, and Identification of a Novel Peptide QFSLP Alleviates LPS-Induced Inflammation
Source: Foods. 2026 Aug 19;15(16):2904. doi: 10.3390/foods15162904 (PMC13512725; doi:10.3390/foods15162904)
Supplement: Supplementary file 1 [file foods-15-02904-s001.zip › foods-4426295-supplementary.pdf]

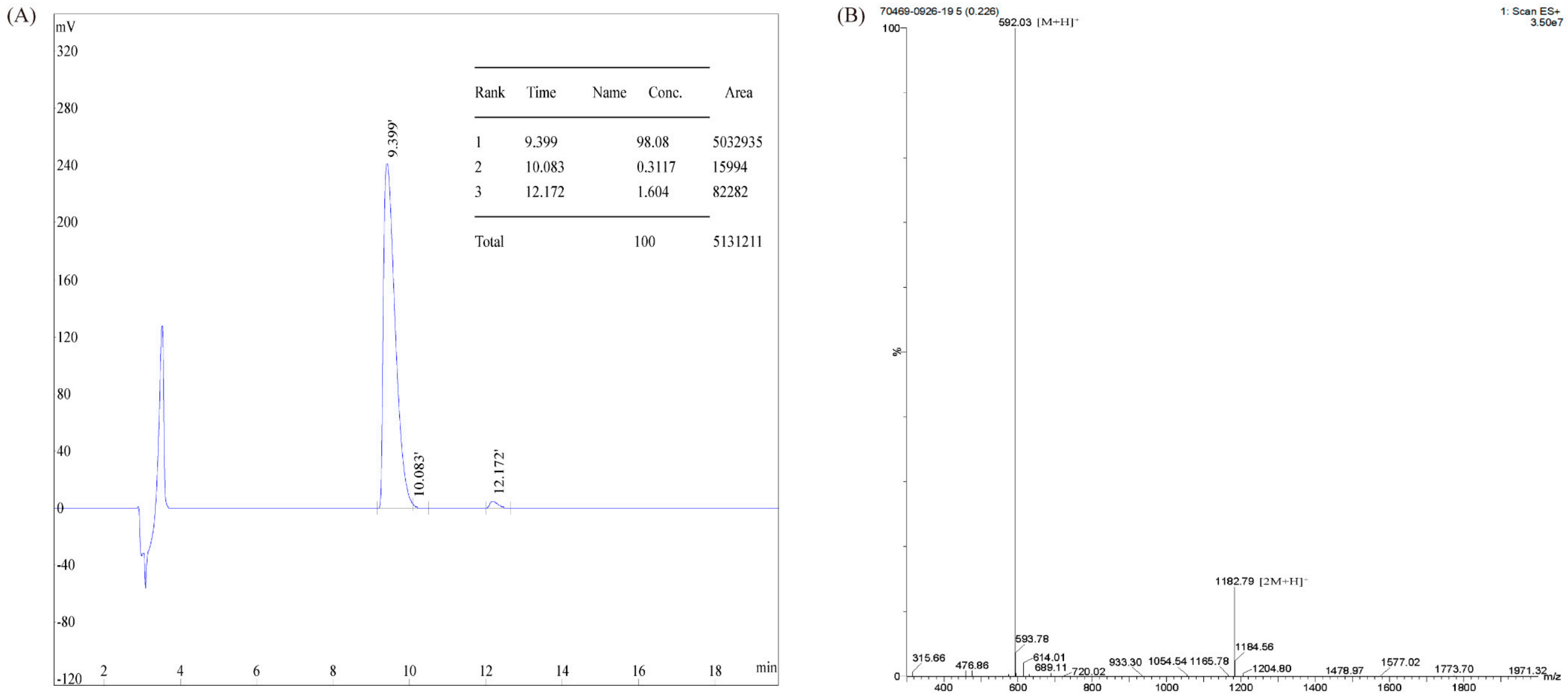

Figure S1 The HPLC chromatogram (A) and mass spectrum (B) of the synthetic QFSLP peptide.

Table S1 Analysis results of physicochemical parameters for 175 Q-containing peptide sequences

No.	Peptide Sequences	Net charge	Boman index	Instability index	Aliphatic index	Isoeletric point	Hydrophobicity	Hmoment α-helix	Hmoment β-sheet
1	QFLPF	-0.003	-1.068	69.960	78.000	5.525	0.860	0.365	0.336
2	QLLPF	-0.003	-1.456	85.040	156.000	5.525	1.060	0.342	0.329
3	TQFLP	-0.003	0.042	16.360	78.000	5.525	0.160	0.425	0.236
4	QLFP	-0.003	-0.590	55.650	97.500	5.525	0.375	0.540	0.260
5	IFPQ	-0.003	-0.590	103.800	97.500	5.525	0.550	0.493	0.220
6	IIPQ	-0.003	-1.075	48.450	195.000	5.525	0.975	0.535	0.173
7	LALPQ	-0.003	-1.222	85.040	176.000	5.525	0.860	0.150	0.236
8	LTQPQLLP	-0.003	-0.139	71.550	146.250	5.525	0.062	0.086	0.209
9	QLPVL	-0.003	-1.668	85.040	214.000	5.525	1.340	0.155	0.423
10	IFLPQ	-0.003	-1.456	85.040	156.000	5.525	1.200	0.089	0.219
11	QLVLP	-0.003	-1.668	46.520	214.000	5.525	1.340	0.287	0.320
12	QFLPA	-0.003	-0.834	69.960	98.000	5.525	0.660	0.375	0.271
13	QLLPF	-0.003	-1.456	85.040	156.000	5.525	1.060	0.342	0.329
14	QLLPF	-0.003	-1.456	85.040	156.000	5.525	1.060	0.342	0.329
15	QIPY	-0.004	0.190	0.300	97.500	5.524	-0.475	0.383	0.568
16	LPFQ	-0.003	-0.590	103.800	97.500	5.525	0.375	0.168	0.678
17	QIVGAP	-0.003	-1.028	-2.017	130.000	5.525	0.833	0.303	0.244
18	HLLGQ	0.088	-0.116	8.000	156.000	6.741	0.100	0.402	0.270
19	ILQPY	-0.004	-0.832	150.240	156.000	5.524	0.380	0.530	0.162
20	QLQPI	-0.003	0.248	111.720	156.000	5.525	-0.060	0.459	0.469
21	IVQPY	-0.004	-0.656	29.540	136.000	5.524	0.460	0.532	0.164

22	QQFLP	-0.003	0.636	69.960	78.000	5.525	-0.400	0.481	0.087
23	LGPQP	-0.003	-0.064	85.040	78.000	5.525	-0.660	0.271	0.276
24	QLPL	-0.003	-1.075	55.650	195.000	5.525	0.625	0.156	0.645
25	TIIPQ	-0.003	-0.346	40.760	156.000	5.525	0.640	0.438	0.145
26	QYPL	-0.004	0.190	19.500	97.500	5.524	-0.650	0.213	0.448
27	LQINP	-0.003	0.468	67.440	156.000	5.525	-0.060	0.136	0.773
28	LIGVQ	-0.003	-1.856	8.000	214.000	5.525	1.720	0.077	0.403
29	QQPIIGGA	-0.003	-0.306	61.713	110.000	5.525	0.175	0.220	0.015
30	SSALLQ	-0.003	0.115	94.767	146.667	5.525	0.717	0.287	0.237
31	QYIPS	-0.004	0.832	25.680	78.000	5.524	-0.540	0.462	0.107
32	ENPILQPY	-1.002	1.161	94.050	97.500	4.000	-0.838	0.328	0.132
33	SFQLP	-0.003	0.208	46.520	78.000	5.525	0.140	0.241	0.604
34	QVPL	-0.003	-0.855	36.800	170.000	5.525	0.725	0.157	0.650
35	QLPF	-0.003	-0.590	103.800	97.500	5.525	0.375	0.138	0.673
36	YQQPIIGGA	-0.004	-0.257	55.967	97.778	5.524	0.011	0.218	0.042
37	QISPL	-0.003	-0.180	95.880	156.000	5.525	0.480	0.400	0.438
38	QILLP	-0.003	-1.844	85.040	234.000	5.525	1.400	0.294	0.387
39	LFPIQ	-0.003	-1.456	46.520	156.000	5.525	1.200	0.198	0.499
40	RQPQCSPL	0.952	2.900	126.475	48.750	8.250	-1.150	0.214	0.136
41	QHIIGGAL	0.088	-1.031	68.488	158.750	6.741	0.888	0.306	0.081
42	QNLYP	-0.004	1.480	32.680	78.000	5.524	-1.220	0.392	0.205
43	LRQPQCSP	0.952	2.900	150.550	48.750	8.250	-1.150	0.382	0.379
44	ENPALQPY	-1.002	1.550	94.050	61.250	4.000	-1.175	0.234	0.203
45	LPFYPQ	-0.004	-0.370	179.533	65.000	5.524	-0.233	0.211	0.361
46	LPFYPQ	-0.004	-0.370	179.533	65.000	5.524	-0.233	0.211	0.361

47	LLPFYPQ	-0.004	-1.020	155.314	111.429	5.524	0.343	0.302	0.274
48	QLAPI	-0.003	-1.222	46.520	176.000	5.525	1.000	0.338	0.351
49	AFLTQ	-0.003	-0.320	-7.080	98.000	5.525	0.840	0.259	0.111
50	QVLP	-0.003	-0.855	36.800	170.000	5.525	0.725	0.515	0.283
51	QLLP	-0.003	-1.075	55.650	195.000	5.525	0.625	0.513	0.279
52	SVLQQPIAQ	-0.003	0.481	87.911	130.000	5.525	0.156	0.341	0.087
53	LLPFYPQ	-0.004	-1.020	155.314	111.429	5.524	0.343	0.302	0.274
54	QSFPI	-0.003	0.208	134.400	78.000	5.525	0.280	0.169	0.424
55	QLPFS	-0.003	0.208	85.040	78.000	5.525	0.140	0.141	0.562
56	ISPFQ	-0.003	0.208	134.400	78.000	5.525	0.280	0.437	0.390
57	LSIPQ	-0.003	-0.180	40.760	156.000	5.525	0.480	0.305	0.416
58	QQILLPF	-0.003	-0.951	118.629	167.143	5.525	0.900	0.405	0.317
59	FLPQ	-0.003	-0.590	103.800	97.500	5.525	0.375	0.450	0.210
60	LGVLQ	-0.003	-1.856	73.200	214.000	5.525	1.580	0.274	0.288
61	QYQLPS	-0.004	1.617	59.967	65.000	5.524	-1.150	0.130	0.435
62	QQLLP	-0.003	0.248	85.040	156.000	5.525	-0.200	0.461	0.061
63	QQLPL	-0.003	0.248	85.040	156.000	5.525	-0.200	0.207	0.421
64	SFLQ	-0.003	0.260	89.000	97.500	5.525	0.575	0.595	0.253
65	QLGAI	-0.003	-1.410	-8.980	176.000	5.525	1.240	0.247	0.346
66	QLLQ	-0.003	0.310	89.000	195.000	5.525	0.150	0.709	0.305
67	QQILLP	-0.003	-0.613	104.633	195.000	5.525	0.583	0.275	0.228
68	TQPQLLPF	-0.003	0.104	95.625	97.500	5.525	-0.063	0.118	0.316
69	QSLFP	-0.003	0.208	134.400	78.000	5.525	0.140	0.374	0.095
70	QQQLLPF	-0.003	0.543	118.629	111.429	5.525	-0.243	0.270	0.257
71	TVLPQ	-0.003	-0.170	85.040	136.000	5.525	0.440	0.365	0.156

72	PTIPQ	-0.003	0.638	40.760	78.000	5.525	-0.580	0.423	0.203
73	QLFLP	-0.003	-1.456	46.520	156.000	5.525	1.060	0.309	0.299
74	QLGIP	-0.003	-1.048	-14.740	156.000	5.525	0.560	0.148	0.485
75	PFIYPQ	-0.004	-0.370	93.100	65.000	5.524	-0.117	0.085	0.204
76	QFLPT	-0.003	0.042	31.440	78.000	5.525	0.160	0.428	0.246
77	QQLLPF	-0.003	-0.290	104.633	130.000	5.525	0.300	0.394	0.183
78	QSFIP	-0.003	0.208	90.120	78.000	5.525	0.280	0.411	0.101
79	GVGSTPILGQ	-0.003	-0.519	19.770	107.000	5.525	0.470	0.086	0.138
80	YQQPIIGG	-0.004	-0.063	72.325	97.500	5.524	-0.213	0.197	0.123
81	QLLPL	-0.003	-1.844	46.520	234.000	5.525	1.260	0.342	0.311
82	SFQQS	-0.003	2.980	134.400	0.000	5.525	-1.160	0.418	0.321
83	QLPPT	-0.003	0.638	85.040	78.000	5.525	-0.720	0.284	0.375
84	QVEPL	-1.001	0.678	31.440	136.000	4.000	-0.120	0.393	0.462
85	QNLLLP	-0.003	-0.430	40.433	195.000	5.525	0.467	0.228	0.171
86	QLLNPL	-0.003	-0.430	3.533	195.000	5.525	0.467	0.631	0.074
87	QVSFI	-0.003	-0.600	-7.080	136.000	5.525	1.440	0.251	0.473
88	QHLPL	0.088	0.072	46.520	156.000	6.741	-0.140	0.184	0.364
89	FYPQ	-0.004	0.675	168.000	0.000	5.524	-0.900	0.282	0.408
90	QHFLP	0.088	0.460	25.780	78.000	6.741	-0.340	0.416	0.025
91	QSLLP	-0.003	-0.180	134.400	156.000	5.525	0.340	0.365	0.076
92	QQPIVGGA	-0.003	-0.196	40.488	97.500	5.525	0.138	0.229	0.033
93	QILPQ	-0.003	0.248	123.560	156.000	5.525	-0.060	0.540	0.341
94	QLLQP	-0.003	0.248	111.720	156.000	5.525	-0.200	0.559	0.262
95	QLQPAS	-0.003	1.292	126.867	81.667	5.525	-0.633	0.263	0.415
96	SFLQQ	-0.003	1.316	111.720	78.000	5.525	-0.240	0.531	0.054

97	ENPIVQPY	-1.002	1.271	18.613	85.000	4.000	-0.788	0.328	0.134
98	LYPPQ	-0.004	0.152	109.720	78.000	5.524	-0.840	0.107	0.236
99	QFSLP	-0.003	0.208	31.440	78.000	5.525	0.140	0.095	0.586
100	QQPIIGGAL	-0.003	-0.819	55.967	141.111	5.525	0.578	0.132	0.130
101	GIEHPIVQ	-0.91	0.274	29.425	133.750	5.240	0.125	0.346	0.268
102	QQLPF	-0.003	0.636	123.560	78.000	5.525	-0.400	0.181	0.447
103	GQTPVFPR	0.997	1.884	47.475	36.250	9.750	-0.663	0.276	0.228
104	QQFLPA	-0.003	0.228	92.067	81.667	5.525	-0.033	0.396	0.097
105	QQFLPA	-0.003	0.228	92.067	81.667	5.525	-0.033	0.396	0.097
106	QFLPAL	-0.003	-1.515	59.967	146.667	5.525	1.183	0.489	0.160
107	QQLLPF	-0.003	-0.290	104.633	130.000	5.525	0.300	0.394	0.183
108	QQLLPF	-0.003	-0.290	104.633	130.000	5.525	0.300	0.394	0.183
109	QQLIQD	-1.003	2.583	72.533	130.000	3.800	-0.950	0.608	0.048
110	QFMP	-0.003	0.053	98.500	0.000	5.525	-0.100	0.452	0.385
111	QPTPQ	-0.003	2.730	85.040	0.000	5.525	-2.180	0.301	0.316
112	QYPTQPPQ	-0.004	2.416	77.550	0.000	5.524	-2.163	0.329	0.229
113	AGFFQ	-0.003	-0.634	8.000	20.000	5.525	0.700	0.328	0.262
114	QQIVPA	-0.003	0.052	90.483	130.000	5.525	0.317	0.426	0.107
115	IYPQ	-0.004	0.190	86.500	97.500	5.524	-0.475	0.310	0.451
116	LTLQ	-0.003	-0.433	89.000	195.000	5.525	0.850	0.120	0.687
117	TLLQ	-0.003	-0.433	89.000	195.000	5.525	0.850	0.545	0.268
118	SFLTQ	-0.003	0.722	-7.080	78.000	5.525	0.320	0.416	0.160
119	LGFPQ	-0.003	-0.660	85.040	78.000	5.525	0.220	0.187	0.271
120	FLTQ	-0.003	0.053	-11.350	97.500	5.525	0.600	0.476	0.168
121	QLIEF	-1.001	-0.094	95.880	156.000	4.000	0.820	0.533	0.469

122	HLTLQ	0.088	0.586	73.200	156.000	6.741	0.040	0.154	0.603
123	QQQQFLPA	-0.003	1.556	119.700	61.250	5.525	-0.900	0.169	0.077
124	QQHIIGGA	0.088	0.276	92.563	110.000	6.741	-0.025	0.241	0.069
125	NSPAYLQ	-0.004	1.284	145.429	70.000	5.524	-0.729	0.159	0.276
126	QVPAL	-0.003	-1.046	69.960	156.000	5.525	0.940	0.233	0.382
127	QVPLA	-0.003	-1.046	31.440	156.000	5.525	0.940	0.105	0.455
128	TFIPQ	-0.003	0.042	65.440	78.000	5.525	0.300	0.429	0.112
129	QLLPY	-0.004	-0.832	46.520	156.000	5.524	0.240	0.384	0.230
130	FALQ	-0.003	-1.043	89.000	122.500	5.525	1.225	0.260	0.551
131	LPFNQ	-0.003	0.856	69.960	78.000	5.525	-0.400	0.226	0.435
132	QQPIVGGAI	-0.003	-0.721	37.100	130.000	5.525	0.622	0.109	0.162
133	VGSPFLGQ	-0.003	-0.610	77.138	85.000	5.525	0.513	0.222	0.089
134	QLPFM	-0.003	-0.942	85.040	78.000	5.525	0.680	0.078	0.468
135	FLQQPSAL	-0.003	-0.019	121.725	110.000	5.525	0.350	0.320	0.057
136	LFQQSPAL	-0.003	-0.019	166.750	110.000	5.525	0.350	0.263	0.086
137	PLTTPQQ	-0.003	1.614	63.600	55.714	5.525	-1.114	0.253	0.087
138	PLQNYP	-0.004	1.233	83.233	65.000	5.524	-1.283	0.383	0.179
139	LRQQAL	0.997	2.392	104.633	146.667	9.750	-0.350	0.264	0.593
140	LSPIQ	-0.003	-0.180	95.880	156.000	5.525	0.480	0.442	0.391
141	QQQLLP	-0.003	1.130	104.633	130.000	5.525	-0.750	0.253	0.176
142	QFMPV	-0.003	-0.766	119.320	58.000	5.525	0.760	0.345	0.332
143	QFLP	-0.003	-0.590	36.800	97.500	5.525	0.375	0.528	0.307
144	QSLAL	-0.003	-0.542	95.880	176.000	5.525	1.020	0.160	0.250
145	LATQV	-0.003	-0.532	-22.160	156.000	5.525	1.120	0.498	0.376
146	AHLTLQ	0.088	0.187	48.517	146.667	6.741	0.333	0.212	0.553

147	LLLGQ	-0.003	-2.032	8.000	234.000	5.525	1.500	0.120	0.211
148	LPFLQ	-0.003	-1.456	150.240	156.000	5.525	1.060	0.353	0.314
149	YQQPIIG	-0.004	0.063	63.600	111.429	5.524	-0.186	0.178	0.084
150	QQQFLPA	-0.003	0.987	107.857	70.000	5.525	-0.529	0.249	0.202
151	QLPAF	-0.003	-0.834	85.040	98.000	5.525	0.660	0.247	0.378
152	LQQPIVGG	-0.003	-0.585	91.850	133.750	5.525	0.388	0.316	0.213
153	QSLPA	-0.003	0.442	172.920	98.000	5.525	-0.060	0.261	0.252
154	QILLPL	-0.003	-2.357	72.533	260.000	5.525	1.800	0.404	0.388
155	LGYPQ	-0.004	-0.036	54.220	78.000	5.524	-0.600	0.060	0.223
156	VAYPQ	-0.004	-0.034	71.200	78.000	5.524	-0.080	0.038	0.220
157	QLQLP	-0.003	0.248	111.720	156.000	5.525	-0.200	0.125	0.692
158	SFQLQ	-0.003	1.316	73.200	78.000	5.525	-0.240	0.058	0.755
159	LLLQ	-0.003	-2.305	89.000	292.500	5.525	1.975	0.380	0.419
160	QYLP	-0.004	0.190	36.800	97.500	5.524	-0.650	0.461	0.149
161	LLQPS	-0.003	-0.180	150.240	156.000	5.525	0.340	0.390	0.274
162	LVLQ	-0.003	-2.085	89.000	267.500	5.525	2.075	0.385	0.414
163	QHIIGGA	0.088	-0.476	76.843	125.714	6.741	0.471	0.373	0.063
164	QLELP	-1.001	0.502	46.520	156.000	4.000	-0.200	0.107	0.670
165	QQQFLP	-0.003	1.453	92.067	65.000	5.525	-0.917	0.272	0.192
166	QHLLP	0.088	0.072	46.520	156.000	6.741	-0.140	0.394	0.034
167	QQLLNPL	-0.003	0.423	31.971	167.143	5.525	-0.100	0.601	0.060
168	QQHIIGGAL	0.088	-0.301	83.389	141.111	6.741	0.400	0.178	0.165
169	TQQQLLPF	-0.003	0.796	95.625	97.500	5.525	-0.300	0.231	0.219
170	LLQKQ	0.997	1.358	120.560	156.000	8.750	-0.660	0.421	0.089
171	QAGLP	-0.003	-0.426	46.520	98.000	5.525	0.020	0.176	0.345

172	QQLP	-0.003	1.540	103.800	97.500	5.525	-1.200	0.523	0.279
173	QALLP	-0.003	-1.222	46.520	176.000	5.525	0.860	0.292	0.235
174	LLAQP	-0.003	-1.222	46.520	176.000	5.525	0.860	0.329	0.266
175	TFLTQPQ	-0.003	1.189	70.457	55.714	5.525	-0.486	0.278	0.196
